# PhenCards: a data resource linking human phenotype information to biomedical knowledge

**DOI:** 10.1186/s13073-021-00909-8

**Published:** 2021-05-25

**Authors:** James M. Havrilla, Cong Liu, Xiangchen Dong, Chunhua Weng, Kai Wang

**Affiliations:** 1 Raymond G. Perelman Center for Cellular and Molecular Therapeutics, Children’s Hospital of Philadelphia, Philadelphia, PA 19104 USA; 2 Department of Biomedical Informatics, Columbia University Irving Medical Center, New York, NY 10032 USA; 3 Department of Pathology and Laboratory Medicine, University of Pennsylvania Perelman School of Medicine, Philadelphia, PA 19104 USA

**Keywords:** Disease, Phenotype, Genetics, Natural Language Processing, Mendelian diseases, Rare disease, Common disease, Drug targets, Collaborative support

## Abstract

We present PhenCards (https://phencards.org), a database and web server intended as a one-stop shop for previously disconnected biomedical knowledge related to human clinical phenotypes. Users can query human phenotype terms or clinical notes. PhenCards obtains relevant disease/phenotype prevalence and co-occurrence, drug, procedural, pathway, literature, grant, and collaborator data. PhenCards recommends the most probable genetic diseases and candidate genes based on phenotype terms from clinical notes. PhenCards facilitates exploration of phenotype, e.g., which drugs cause or are prescribed for patient symptoms, which genes likely cause specific symptoms, and which comorbidities co-occur with phenotypes.

**Supplementary Information:**

The online version contains supplementary material available at 10.1186/s13073-021-00909-8.

## Background

Phenotypes describe any observable traits: common traits, such as hair color, or rarer traits like craniosynostosis (a birth defect in which the bones in a baby’s skull join together too early). To understand more deeply how genetic mutations can discriminate between subclasses of phenotypes, researchers have made efforts to use phenotypic trait information to understand frequencies of phenotype occurrence in conjunction with disease and genetic mutation data [1–4]. In addition, new links between genetic pathways, expression data, and phenotype [5–7] are slowly pushing the field in a direction where researchers and clinicians can provide insights into how genetic mutations influence different pathways in the body, and how these effects are manifested in the phenotype. In recent years, several studies have shown the utility of using clinical phenotype information to facilitate gene identification from clinical genome and exome sequencing data [8–12].

Phenotype vocabularies are superb tools for facilitating the investigation and classification of genetic diseases. Copious web servers, databases, and other resources for phenotypic terms and diseases exist: HPO (Human Phenotype Ontology) [4, 13], MeSH (Medical Subject Headings) [14], OHDSI (Observational Health Data Sciences and Informatics) [15], ICD-10 (International Classification of Diseases, version 10) [16], UMLS (Unified Medical Language System) [17, 18], Disease Ontology [19, 20], OMIM (Online Mendelian Inheritance in Man) [3, 21], DECIPHER (DatabasE of Chromosomal Imbalance and Phenotype in Humans using Ensembl Resource) [22], and Orphanet [2]. However, few of them link related biomedical information to disease and phenotypic terms, and there are numerous gaps in information. Pathway data is similarly limited to specific disease terminology [5–7]. Most phenotypic resources such as HPO or Malacards [23] are limited in their queries, as their data can only access disease-specific or ontology-specific aspects of phenotype information, without comprehensive query functionality that allows one to link all phenotype terminologies to vital biomedical information including drug, gene, pathway, disease, grant, physician, literature, and clinical trial data. There are portions of this data contained in each resource, but none have access to all of it. Ideally there should be one place where clinicians and researchers can go to and find a comprehensive collection of information about a clinical phenotype. Without this singular data resource, what clinicians, researchers and genetic counselors can ascertain about clinical phenotypes is limited to their personal awareness of individual tools and databases.

To fill this void, we have created PhenCards (https://phencards.org), a data resource and a search engine for linking relevant biomedical knowledge to human clinical phenotype terms. PhenCards is among the first resources of its kind, striving to link all possible biomedical knowledge to human phenotypes. We incorporate all of the aforecited databases, drug, procedural study, surgery, pathway, funding opportunity, literature, and hospital-wide patient term co-occurrence data, as well as relevant gene information for extracted HPO terms. Providing all of this data in a single web server will allow clinicians, researchers, and genetic counselors to add new layers of information to previously limited genetic studies and make more informed clinical decisions.

## Construction and content

### Implementation and resources

The entire site uses HTML, Jinja2, CSS, Javascript, jQuery, and Bootstrap 4 to create the look, implement the autocompletion, and display the data on the frontend. The backend uses docker-compose [24], Elasticsearch [25], Python 3.8, Flask [26], the Apache HTTP Server [27], and Certbot with Let’s Encrypt [28]. Several python packages are utilized including Elasticsearch, Ray [29], and Beautiful Soup. The site has been tested and shown to work on Windows/macOS/Linux Chrome, Opera, Edge, and Safari, iOS Safari/Chrome, and Android Chrome. The index page is easy and straightforward to use with explanations on input. Highly detailed frontend, backend, secure query, and literature search implementations, as well as how to use all site resources, are described in Additional file 1 and Additional file 2. We also possess numerous resources, some of which are real-time and some of which require regular updates (Table 1). Further information about the licenses and versions are shown in Additional file 1.

**Table 1:** Resources in PhenCards. Some resources require regular updates and downloads to stay abreast of changes, but the majority of resources are API or web-based and require no changes to stay up-to-date

Resource(s)	Method of access	Update needed?	Content
HPO [4, 13] (includes OMIM [3, 21] and Orphanet [2]), Disease Ontology [19, 20]	Elasticsearch [25] on indexed database	Yes, monthly	Standardized phenotype and disease terms
ICD-10 [16], UMLS [17, 18], OHDSI ATHENA [15], MeSH [14]	Elasticsearch on indexed database	Yes, yearly	Standardized phenotype and disease terms
Pharos (disease) [30]	API	No	Disease aliases, expression, drug, pathway, Gene Ontology data
IRS (Internal Revenue Service), Open990	Elasticsearch on indexed database	Yes, yearly	Nonprofit grants and foundations
NIH (National Institute of Health) Federal Reporter, NIH FOAs (funding opportunity announcements)	API	No	Federal grant and projects
Direct2Experts [31]	API	No	Collaborators, specialty physicians
openFDA [32], Tocris, APExBio, Pharos (target) [30], DrugCentral [33]	API	No	Federal and company drug, drug target and adverse effect data
Pathway Commons [34] and KEGG [5, 34]	API	No	Pathways: diseases, biological functions
ClinicalTrials.gov [35]	API	No	Clinical trials: studies, procedures, drugs
Columbia Open Health Data [36]	API	No	Co-occurring patient drug, procedure, and condition terms
Doc2Hpo [37]	API	No	NLP algorithm for optimally extracting terms from text
Phen2Gene [38]	API	No	Algorithm ranking candidate genes for a set of HPO terms
PubMed [39]	API	No	Biomedical literature
Google Scholar	API	No	Large-scale scholarly search engine


**Additional file 2: Supplementary Video** on how to use the website. A tutorial that is also available on YouTube with captions for those hard of hearing at https://www.youtube.com/watch?v=9FT4pFgeA08.

### Basic workflow

**Fig. 1:**
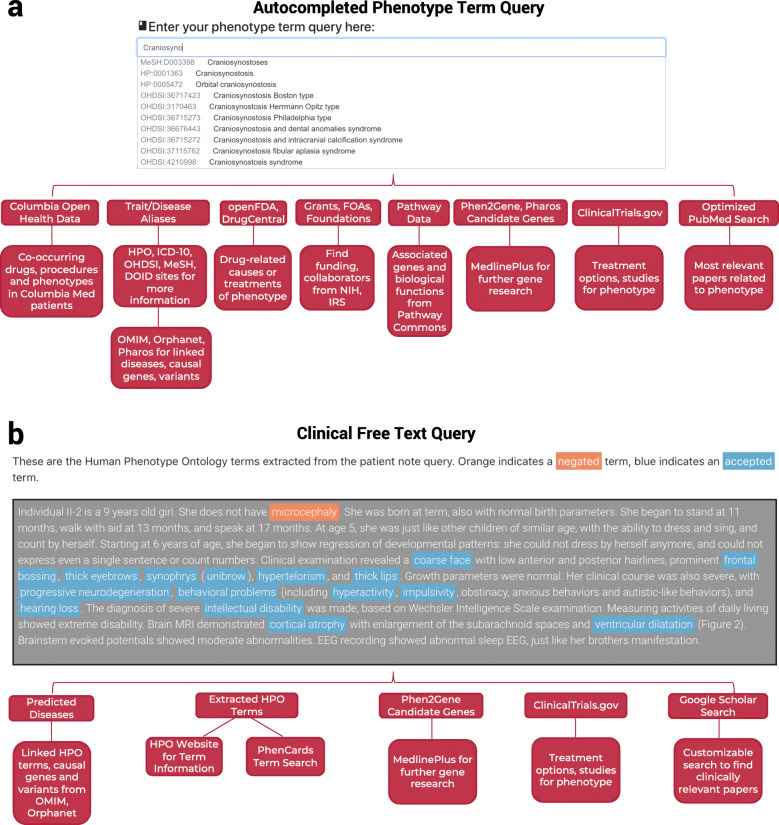
Website workflow. A user queries the website for a phenotype term using a string search, or for multiple extracted phenotype terms using clinical notes extracted from Griffin et al. [43]. **a** If using a phenotype term query, the user has several avenues available: searching databases for term Aliases and Diseases, obtaining candidate genes for the term and exploring gene information, co-occurring terms in Columbia Medical System patients, as well as protein pathway, grant, nonprofit, pathway, literature, and clinical trial and drug data. **b** Using clinical notes to extract phenotype terms, the options are more limited, but still plentiful: clinical trial data, literature search, predicted diseases, candidate genes from Phen2Gene, and exploring extracted terms

After receiving 3 characters of user phenotype term input, the site begins to autocomplete known phenotype terms for the user (though choosing one is not required). After submitting a query, PhenCards makes multitudinous resources available to the user. We first and foremost provide aliases for matching phenotype and disease terms. There are links to each site where applicable for further investigation, and hoverable tooltips that explain all data tables on the site. Pharos [30] information for UniProt [40] disease aliases is expandable in PhenCards to obtain Gene Ontology [41] data, expression data, pathway data, and drug development stage data for the term. For further facilitating genetic studies, PhenCards utilizes Phen2Gene [38] and Pharos again to obtain relevant gene information for extracted HPO terms for user queries, and these genes link out to MedlinePlus [42] and the Pharos site. The Pharos drug target data can be further explored in PhenCards to learn ligands for the target, protein-protein interactions, its expression in certain tissues, and its novelty. We search APExBio, Tocris, DrugCentral [33], and openFDA’s FAERS (FDA Adverse Event Reporting System) database [32] as well as drug indication and adverse effect data, study data, and surgery data from ClinicalTrials.gov [35] for potential treatments for the phenotype, as well as drugs that may have led to the phenotype. PhenCards integrates COHD (Columbia Open Health Data) [36] to find which drugs, conditions, and procedures significantly co-occur with phenotype terms in patients. We have consolidated pathway data for phenotype terms from several places [5, 6, 34]. PhenCards obtains active funding opportunity announcement data from the NIH, and nonprofit foundation and grant data from Open990 and the IRS, and collaborator and physician data from Direct2Experts [31]. Lastly, we have also included a complex literature search for terms using PubMed Entrez Search and Fetch [39] (Fig. 1a). Further details of the site’s implementation and the API queries can be found in Additional file 1.

For users with clinical notes that may describe a de-identified patient’s phenotype using free text, we are able to extract HPO terms using Doc2HPO [37] and link them to ClinicalTrials.gov data and create a custom Google Scholar query. By clicking on the HPO terms, one can investigate more information about the terms on the HPO website or use PhenCards to search for the same information in Fig. 1a. Using all of the HPO terms, we also predict a potential disease phenotype and create a candidate gene list using Phen2Gene all of which can be further investigated through links to the sites of their respective databases (Fig. 1b).

### Disease prediction algorithm

We use HPO terms extracted from clinical notes by the Aho-Corasick algorithm [44] in Doc2HPO in order to find the most likely disease phenotype for a potential patient. Using an exact match in Elasticsearch for linked HPO terms to disease databases such as OMIM and Orphanet, we add the Elasticsearch scores together for each disease linked to each exact HPO term match and the disease with the highest aggregate Elasticsearch score is ranked first. The score itself is arbitrary, the scale only exists to rank the matches against one another. Elasticsearch score is calculated using an algorithm called BM25, which is similar to tf-idf (term frequency–inverse document frequency), except that it accounts for document length (greater details available in Additional file 1).

### Pathway query

First, we query the KEGG Pathway database for diseases related to the phenotype term using the KEGG FIND API query. This searches names but also disease description text. Then using the official KEGG disease names we can find relevant linked pathways using KEGG’s LINK API query. Pathway Commons already does all this by default for Reactome and other databases, but not for KEGG.

### Phen2Gene query

Using the “Phenotype search” query, which is the main function of the site, leads users to the results page. There, the number one HPO term result from the phenotype term query is used to search for the top 1000 candidate genes in Phen2Gene [38]. If using the “Clinical notes” query function of the site, Doc2HPO [37] extracts the non-negated HPO terms and PhenCards uses all of them to query Phen2Gene for the top 1000 candidate genes.

### COHD concept co-occurrence ranking

Patient concepts (conditions, drugs, and procedures) from COHD are derived from the Columbia University Irving Medical Center’s Observational Health Data Sciences and Informatics (OHDSI) database and are in structured OMOP (Observational Medical Outcomes Partnership) format. The p-values for each respective concept pairing’s co-occurrence are based on chi-square tests of their frequencies in the COHD database (further details of calculation in Additional file 1).

## Utility and discussion

### Performing a patient-centered query

**Fig. 2:**
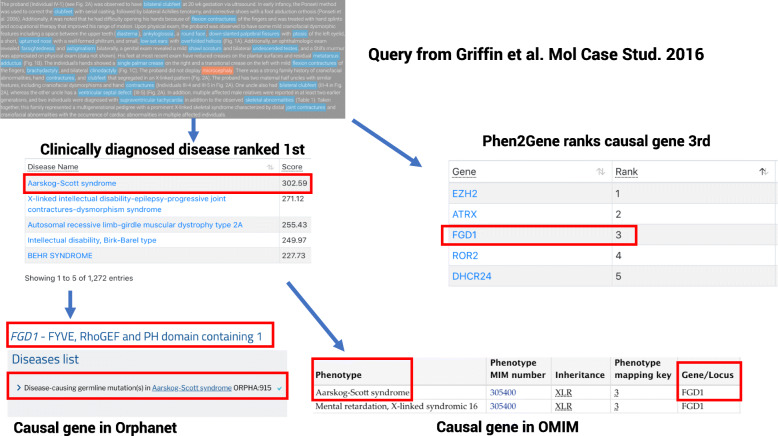
Clinical note query. An example query using clinical notes from a patient provided by Griffin et al. [43] The HPO terms are used to rank potential candidate diseases, and the first ranked disease by amalgamated Elasticsearch score is the diagnosed disease of Aarskog-Scott syndrome. Phen2Gene ranks the causal gene, FGD1, 3rd of all potential candidate genes in the genome using the extracted HPO terms. We can follow the HPO link out to the disease name and from there click on the Orphanet and OMIM links to discover that FGD1 is listed as the causal gene on these sites as well

PhenCards provides a great default example of what a user can investigate using clinical notes in free text format drafted by clinicians or researchers. In this example, these clinical notes come courtesy of Griffin et al., from the section “Clinical Presentation and Family History” [43]. Using these very thorough de-identified clinical notes, we extract several useful HPO terms, which rank the actual disease diagnosis of Aarskog-Scott syndrome as the first result in our disease prediction algorithm (Fig. 2). As the disease name is identical in both OMIM and Orphanet, by clicking the disease name link out to HPO for external navigation, we can see the causal gene identified in Griffin et al., FGD1, is listed as the causal gene for Aarskog-Scott syndrome on both sites. Additionally, FGD1 is ranked 3rd by Phen2Gene. If a user had these notes for this patient as well as variant data for the patient, this could rank the gene even higher by only accepting the genes with overlapping variant data as potential candidates. The algorithms are fairly robust for a large number of HPO terms, as 5 terms were arbitrarily removed in 5 different combinations for this example and we still returned Aarskog-Scott syndrome and FGD1 at the same or higher ranks.

FGD1 provides a potential drug target for the patient. If FGD1 has been used previously and proven unsuccessful, the ClinicalTrials.gov search may provide other treatment alternatives, and the customizable literature search in Google Scholar is preloaded with the HPO terms and can be used to search for relevant literature. In fact, we used it to search for several test case studies with clinical free text. The example in Fig. 2 only requires 3 of the terms to be used in our Google Scholar search to obtain the Griffin et al. paper.

### Using phenotype search to investigate a rare symptom

**Fig. 3:**
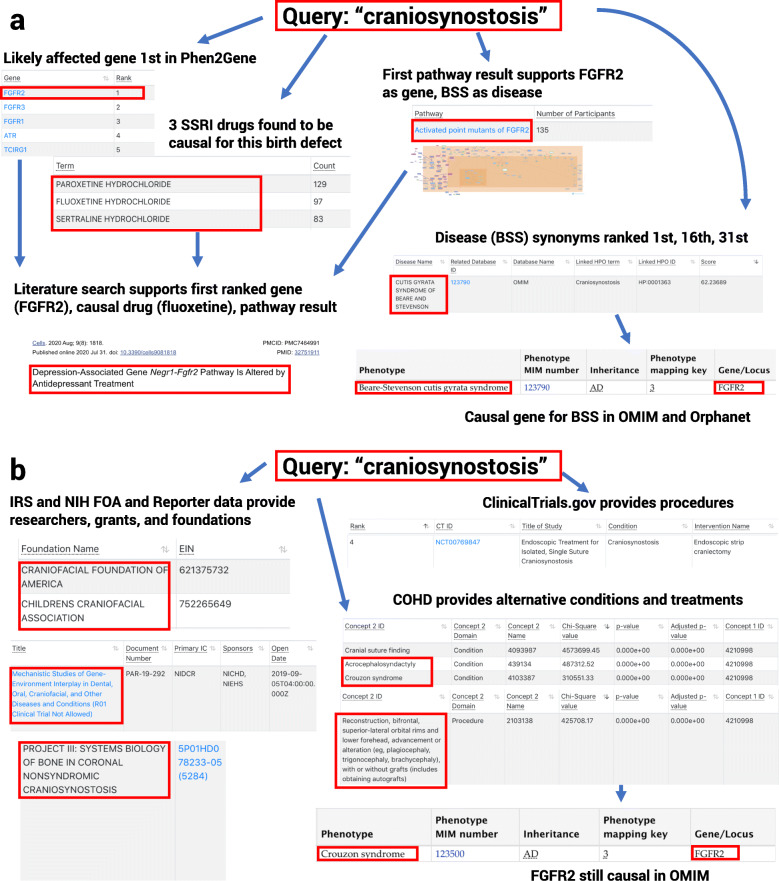
Phenotype term query. An example query using “craniosynostosis” as the searched term. **a** A researcher may wish to know what genes are likely causal for a disease or phenotypic trait. FGFR2 is shown to be the most likely causal gene on OMIM, Orphanet, and Phen2Gene for craniosynostosis, particularly for “Beare-Stevenson Syndrome” (BSS). Pathway information from Reactome for “Activated point mutants of FGFR2” demonstrates further evidence that FGFR2 is the most likely causal gene and provides several alternative drug target candidates. Literature and openFDA data also support fluoxetine, which has significant effects on FGFR2 and its pathways, as a potential cause of the condition. **b** Alternative ways to look at this symptom include finding alternative co-occurring conditions in COHD, in addition to finding past treatments for patients in the Columbia University Irving Medical Center in conjunction with clinical trial data. FGFR2 is still shown to be a causal gene for many alternative syndromes with this condition. Finally, a user can find current funded research, its principal investigators (PIs), new sources of NIH funding, and non-profit foundations that support research and treatment for the condition

The phenotype term search provides access to a myriad of data sources, but its primary use is to aid researchers and genetic counselors in further investigating a patient’s phenotype when they do not have access to clinical notes or curated terms. Using a rare symptom, “craniosynostosis” which only occurs in as few as 0.04% of births [45], we can show how a researcher might narrow down useful information (Fig. 3a). Using the openFDA and DrugCentral FAERS data, we can see that SSRI antidepressants like paroxetine are a common cause of infant craniosynostosis as a birth defect (and from drinking breast milk with the drug) [46]; this birth defect has also been associated with fluoxetine [47] and sertraline [48]. The most common genetic cause of craniosynostosis is mutations in FGFR2, which is the first ranked gene in both the Phen2Gene candidate gene and Pharos drug target results. Beare-Stevenson cutis gyrata syndrome is commonly associated with this symptom and mutations in FGFR2 [49] on OMIM and Orphanet, as is “Craniosynostosis, nonspecific” from OMIM, both of which appear in the Diseases results for this term. Pathway Commons further supports this with the top Reactome hit “Activated point mutants of FGFR2,” where the first mentioned disease entry is Beare-Stevenson cutis gyrata syndrome; this pathway can also be used for alternative potential drug targets. A basic literature search of fluoxetine shows the drug affects one of the FGFR2 sub-pathways, explaining a likely cause of this potential genetic mutation [50].

Relevant surgical procedures and investigational study data can also be found for this condition thanks to COHD and ClinicalTrials.gov data collected by PhenCards. Finally, PhenCards provides several non-profit foundations where one could seek financial help for treating or researching this ailment, including the Craniofacial Foundation of America. There are several relevant active FOAs displayed from the NIH, and funded grants and their PIs’ information from the NIH Federal Reporter service (Fig. 3b). In addition, we provide access to collaborators and physicians via Direct2Experts. Our goal is to arm researchers, genetic counselors, and clinicians with enough knowledge to assist their patients in relieving their ailments, whatever path they choose.

### Discussion and future directions

The PhenCards web server can facilitate biomedical researchers in formulating new hypotheses on specific clinical phenotypes, locating potential funding and financial support, and help clinicians in analyzing clinical texts and derive possible diagnoses. If a clinician has trouble discerning their patient’s phenotype from their notes, our phenotype prediction algorithm can assist in this feat, the extracted terms can be used to rank candidate causal genes, and provide potential sources of treatment by linking clinical trials data. If the disease is too new to have this information in existing databases, we provide the means to supplement this with a literature search involving the extracted terms. For a more specific individual condition, the phenotype term bestows the researcher with a wellspring of knowledge, such as potentially related diseases, prescribed drugs or drugs that caused it, clinical trials specific to the phenotype, pathways involving the condition, causal genes for the condition, and procedures and conditions co-occurring with the phenotype in other patient data. Finally, if the varied information fails to supply the researcher with new hypotheses, it can put them in touch with other researchers, physicians, grants, and foundations that may lend support in the investigation or treatment of the condition.

The current version of the PhenCards server has some limitations. Currently, only the English language is supported, though moving to other languages in the future for some resources is not difficult: UMLS and OHDSI, for example, support several other languages, and Elasticsearch can be configured to allow for multilingual query decomposition. Language translations are already underway by the HPO team who has gathered volunteer groups worldwide to translate and parse the HPO terms. We plan to support the multi-language HPO upgrade, as well as incorporate terminologies that already include more than one language. To further linguistic inclusivity, we are also making efforts to parse clinical notes from other languages using NLP algorithms specialized to those languages and map them to these multi-language terminology sets. In the near future, we aspire to continue adding resources, more drug databases, pathway databases, incorporate complex searching into the patient page, and link more disease information to the search terms. Furthermore, our data on physicians, foundations, grants, and research data is also currently limited to the USA only, but we would appreciate incorporating this data from other countries. For patient co-occurrence data, COHD only reflects the population demographics in one medical institution. PhenCards would benefit greatly from NLP-based question and answer services like “What diseases have seizures?” or “What drugs are linked to conditions with palmar creases?” Lastly, we would relish the idea of incorporating pathology, physical or facial images related to phenotype queries; there are some site scraping and Twitter-based AI bot builders we have begun collaborating with to accomplish this.

## Conclusions

While databases such as HPO have been extremely useful for researchers in genomic medicine, PhenCards (https://phencards.org) adds a new layer of investigational ability for phenotype terminology that did not exist previously, by integrating multiple sources of biomedical knowledge and linking them to presentations of phenotype. When clinical researchers discover a new undiagnosed case, characterizing it by phenotypic traits is always the first step. PhenCards can not only aid in finding similar diseases, but link all relevant information to give researchers the best possible chance of identifying it by comparing it to similar diseases or even supplying novel candidate genes. We sincerely hope that with researcher and community involvement we can add even more useful knowledge to our web server. Our goal is to provide both a one-stop shop and a lasting, continuously updated resource that will allow for novel insight into research of human phenotypes to further our understanding of human health and both rare and common diseases.

## Supplementary Information

**Additional file 1.** Contains Supplementary Methods and Results.

## Data Availability

All data can be accessed through https://phencards.org and its API. The code for creating the site is on https://github.com/WGLab/PhenCards [51] and the data used for constructing the Lucene index with Elasticsearch is on Zenodo at: https://zenodo.org/record/4755959 [52].
