# Supplementary material for: PhenCards: a data resource linking human phenotype information to biomedical knowledge
Source: Genome Med. 2021 May 25;13:91. doi: 10.1186/s13073-021-00909-8 (PMC8147460; doi:10.1186/s13073-021-00909-8)
Supplement: Supplementary file 2 — Additional file 1. Contains Supplementary Methods and Results. [file 13073_2021_909_MOESM1_ESM.docx]

# **SUPPLEMENTARY METHODS**

**Implementation**


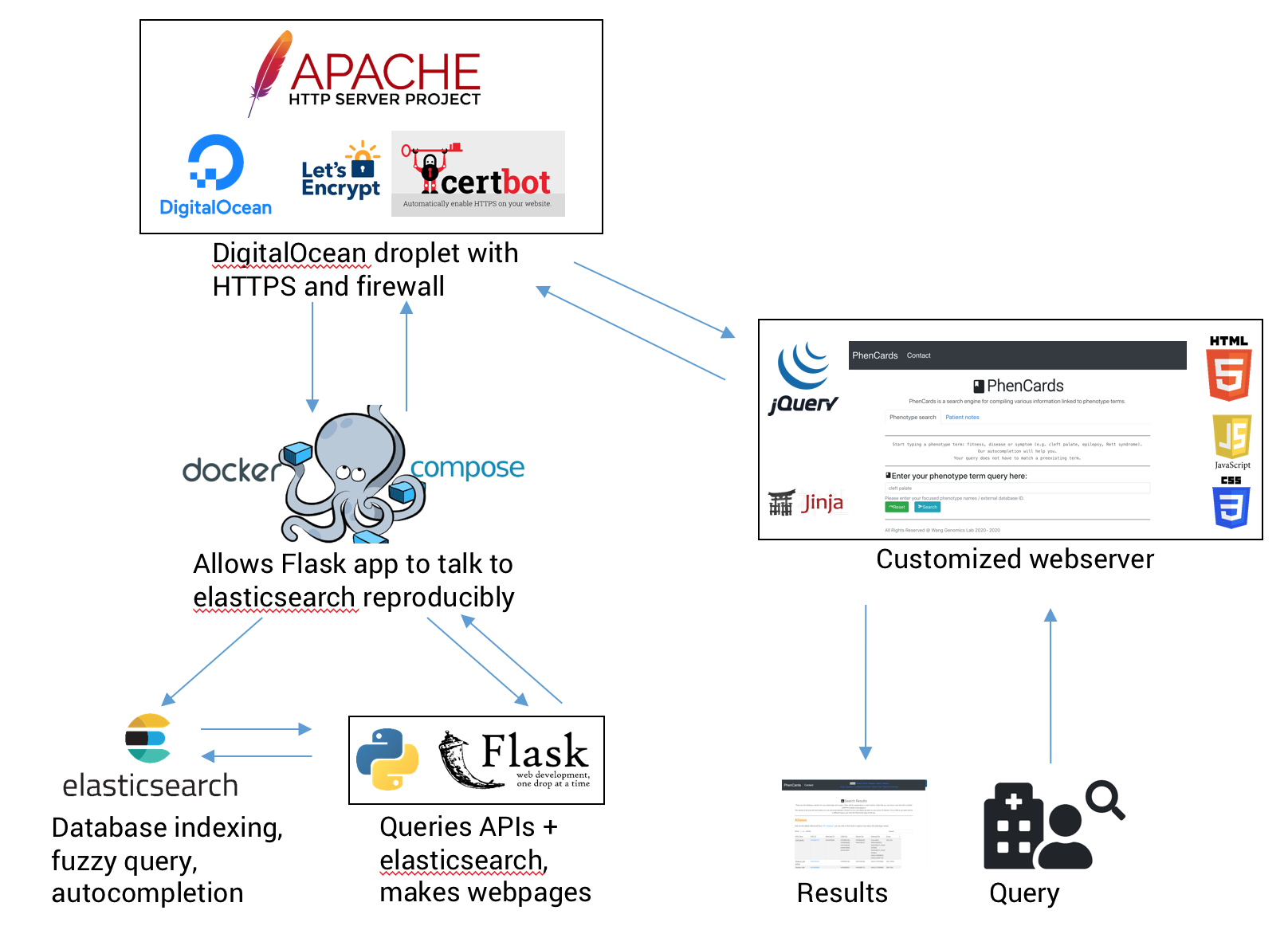


**Figure S1. Website workflow.** A user queries the website for a phenotype term using a string search, or for multiple extracted phenotype terms using clinical notes. The customized website represents a framework to communicate with the Apache server on the DigitalOcean droplet that hosts the backend via HTTPS, secured by certbot. Docker-compose lets the Flask app and Elasticsearch containers communicate back to the frontend for rapid, reproducible deployment. (For Figure 1, I think there is no need to put much details there with logos, especially not digital ocean and certbot apache etc, since they are all copyrighted image and because we may change them in the future; the more important yet less likely changeable things are the actual data flow, in this case, figure 1 should show an example query of a few words of phenotype terms, or a paragraph of clinical notes, and show that the web service can analyze them and generate results with hyperlink to various databases organized by categories, as a workflow that clearly tells readers what are the functionalities).

**Secure queries**

To ensure that patient note data (in case accidentally not deidentified) is not captured by bots or portscan, we used Certbot in conjunction with Let’s Encrypt to ensure that the whole site is HTTPS encrypted and the certificate is kept up-to-date automatically every month. In addition, for the UMLS data queries, which require a UMLS Technology Services login through the NLM (National Laboratory of Medicine), this is now a redirect to the NLM website, and an authentication token is given back to PhenCards via HTTPS to the Flask backend. We do not store any information, hashed or otherwise, we simply obtain a value of true or false and either authenticate or kick the user back to the Results page.

**Building the backend**

We used docker-compose to build the backend. Two self-contained dockerized images are used to run the site: a pre-built image with the most currently up-to-date version of Elasticsearch (7.9) and a Flask app built in Python that communicates with the Elasticsearch container and website APIs using string queries that vary from simply searching the phenotype term, to queries as individualized and complex as searching all diseases or drug targets linked to a term and all information linked to each disease and each target.

Before being indexed by Elasticsearch, the data from the Aliases[1-5], Diseases[6-9], Open990, and IRS 990 AWS databases are mined and parsed and then tokenized into ngrams, with stopwords filtered for fuzzy database querying, which is combined with an unfiltered analyzer to prioritize exact matches. The autocompletion query is then filtered by stopwords, and analyzer in forward and reverse with a shingle ngram token analyzer for fuzzy autocomplete. The autocompletion results have also been prioritized for more commonly used databases like HPO.

The results are returned from Elasticsearch in JSON format and ported to HTML templates which generates the webpages. In addition, the Flask app container communicates with various site APIs from the Related Terms[10], Genes[11], Pathways[12, 13], Clinical Trials[14], Drugs[15], Literature[16] and Foundations and Grants sections as well as the UMLS Authentication API and the Doc2HPO[17] API for clinical note input. This data is passed to the frontend the same way in JSON format.

**Designing the frontend**

The JSON data from the backend is passed to Jinja2 in the HTML templates which is used to rapidly generate HTML tables from a small amount of code. These tables are then turned into jQuery DataTables. The visualizations like the navbar highlighting, smooth scrolling, and rapid table searching are done using jQuery, and Mark.js is used to highlight patient note terms. The site design components like the navbar, tabs, new window link outs and hover tooltips were done using HTML, Bootstrap 4 and custom CSS.

**Custom PubMed literature search**

The PubMed custom search can take a while because it grabs the top 200 results from PubMed sorted by relevance using their Best Match sorting algorithm, and then queries each result to see how many citations there are catalogued in Pubmed and then grabs the top 25 most cited of these top 200 queries. This is because Best Match sorts by more factors than citations, also whether the MeSH terms appear frequently or if it has been trending. The combination of these two sorting methods tends to give informative results, as they are not only old, highly cited papers or query-relevant papers but a combination of the two.

# **SUPPLEMENTARY RESULTS**

**Running a simple query**

In the default tab for the main page, “Phenotype search”, as the user begins typing three or more characters, the autocompletion will begin to suggest possible disease and phenotype terms from the Elasticsearch indices for Aliases and Diseases (**Figure S2a**). Alternatively, the user can click on the “Patient notes” tab to submit deidentified clinical notes (**Figure S2b**). The user need simply press Enter or click the Submit button to be taken to either the Search Results or Patient Results page. The user can also click the Reset Button to clear all text entered.


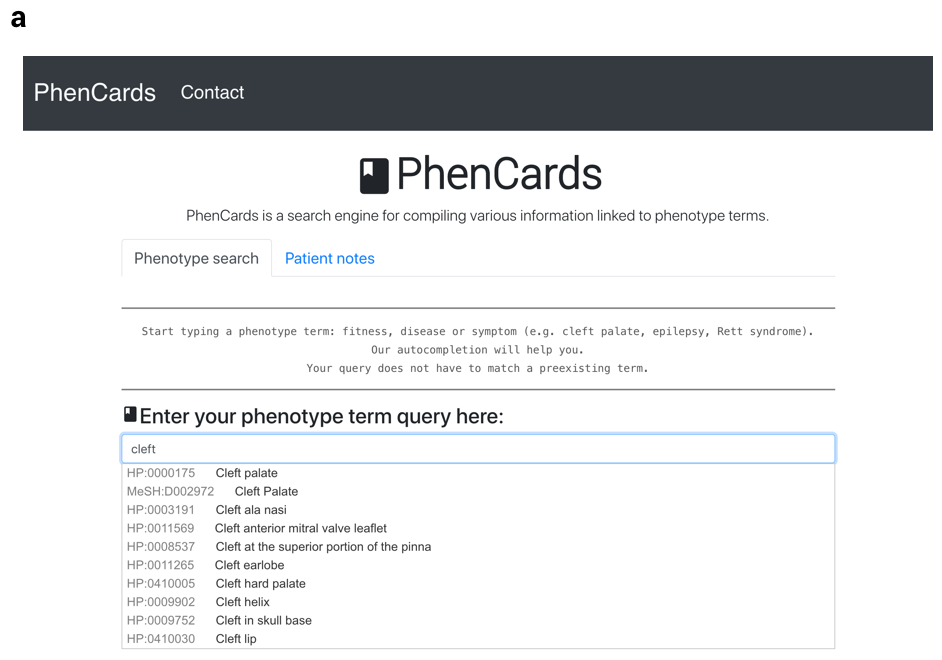

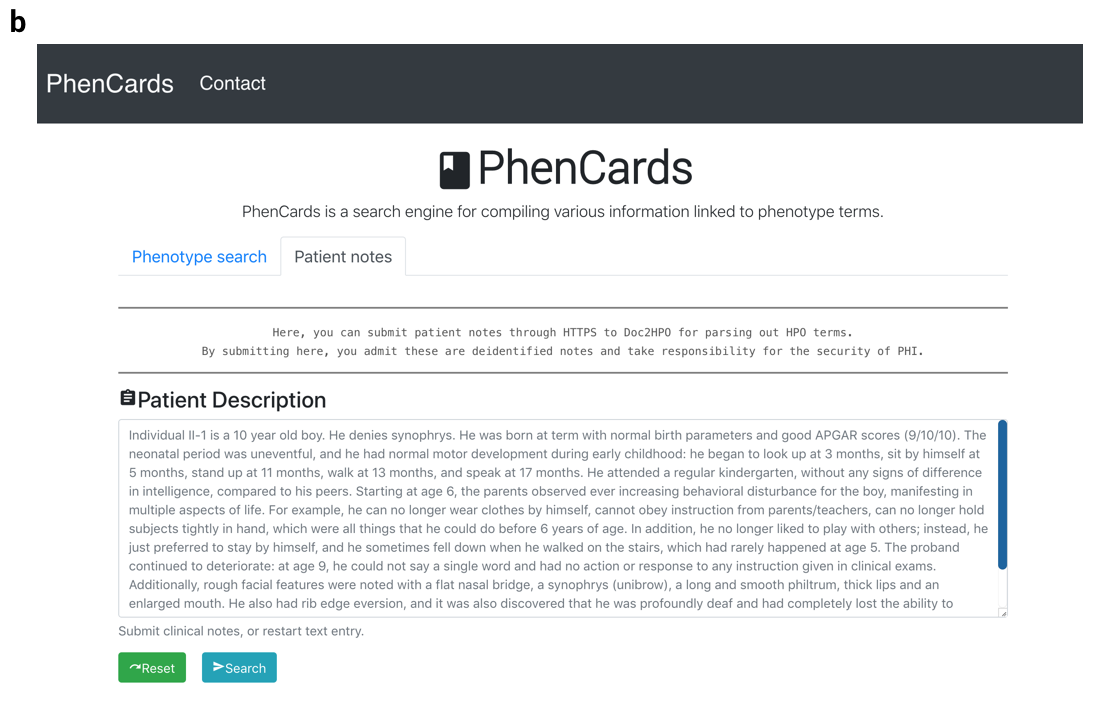


**Figure S2. Basic query and patient note submission examples.**

**Navigation of the results page**

In each of the ten sections indicated by the navbar at the top, there are hoverable tooltips with detailed information about the headers of each data table and what it means. Some data have hovertips within the tables as well, such as the HPO Aliases table (**Figure S3a**). The Aliases section contains data from HPO, OHDSI, MeSH, ICD-10, and UMLS. The convenient navbar at the top enlarges the font size automatically on mobile, sticks to the top so the user can easily navigate, smooth scrolls on click and changes the active section highlights depending on click or scroll location.

We are the only site that currently has searchable UMLS data, most likely because personal UTS license authentication is a necessary step to search the data. As discussed in the Methods section, we have an interface for securely authenticating the UMLS data after clicking on the green “Access UMLS” button which will kick the user back to the Search Results unless they are successfully authenticated by the NLM at their authentication page (**Figure S3b**). There are also links like “Pubmed” or “Pathway Commons” that link out and open a new window as well (but do not require any authentication) because they are API calls that do not use Elasticsearch.


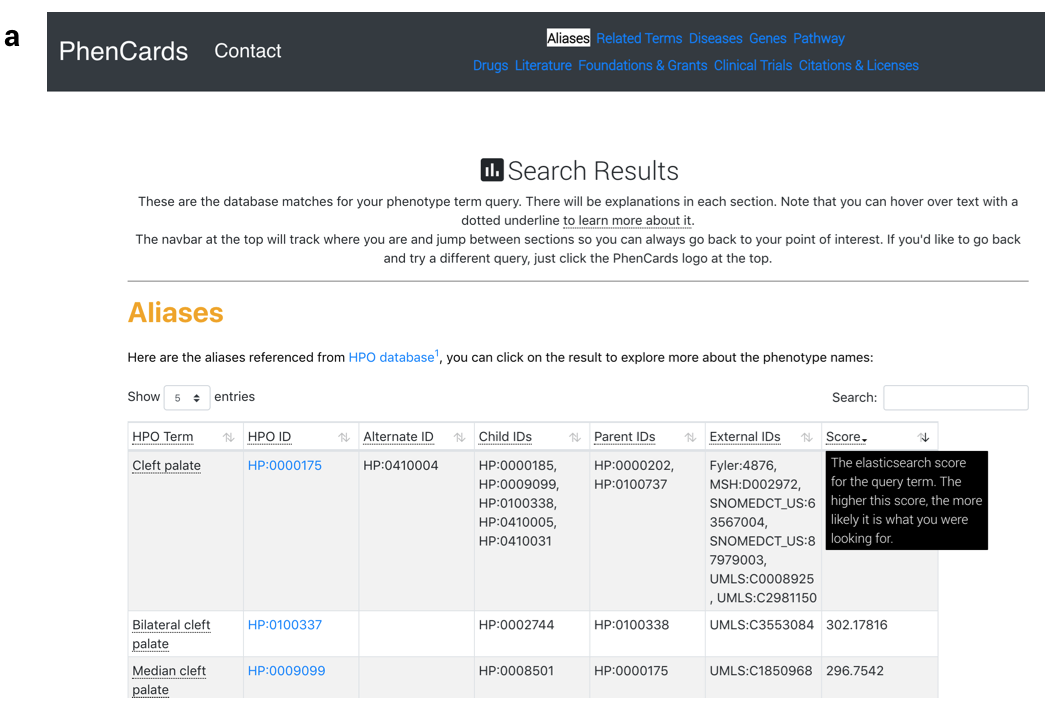


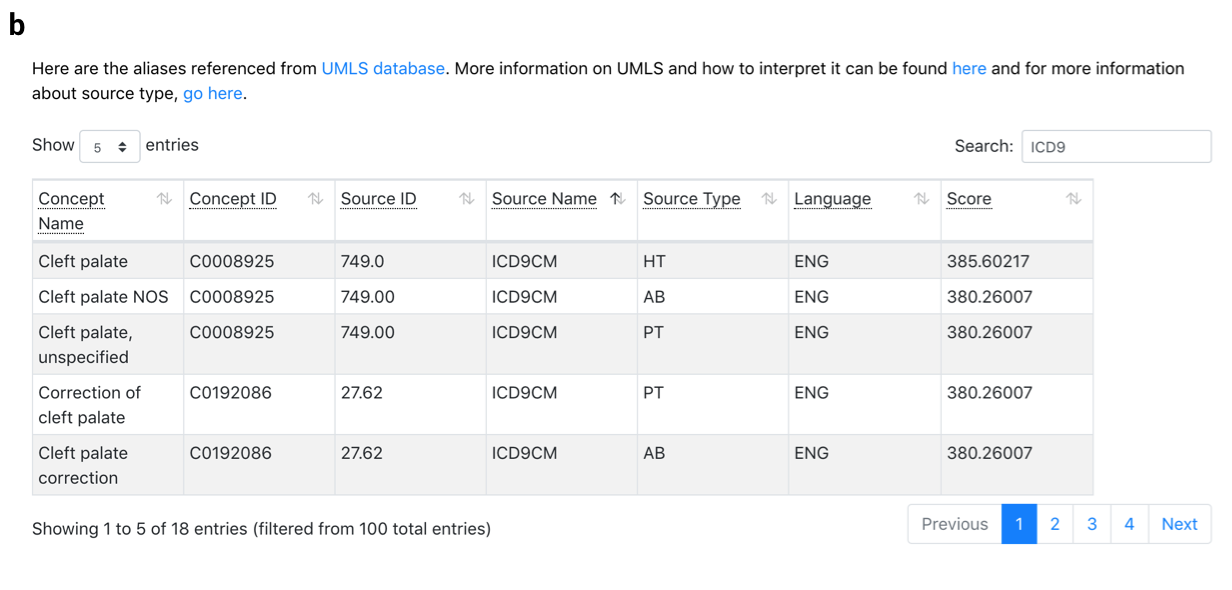


**Figure S3. Navigating the results page for “cleft palate,” and the UMLS login.**

**Digging into related terms with COHD**

Access to the COHD (Columbia Open Health Data) resource is provided in the Related Terms section. Clicking on an OHDSI concept ID links out to our COHD page. For example, using the OHDSI term “Contracture of palmar fascia,” one can see the ancestral OHDSI terms in patients that exist in COHD for the term (**Figure S4a**), as well as — sorted by chi-square significance value — the conditions (**Figure S4b**), drugs (**Figure S4c**), and procedures (**Figure S4d**) that co-occur most significantly with the search term. For “contracture of palmar fascia,” the most common drug seen in patient notes is collagenase from *Clostridium histolyticum[18]*. For the same term, the most common procedure is fasciotomy, also known to be the most common procedure for the synonym condition that is the most co-occurring, Dupuytren's disease[19].

Patient concepts are counted once per patient, not once per note. The chi-square value between two concepts is calculated using a 2x2 contingency table containing four values:

1. The number of patients with the first term and the second term
2. The number of patients without the first term but with the second term
3. The number of patients who have the first term but not the second term
4. The number of patients that don't have either term

The samples must be random, and the observations independent of one another, which they mostly are. The one disadvantage of this calculation is it is artificially inflated by the fourth number, which is often extremely large.


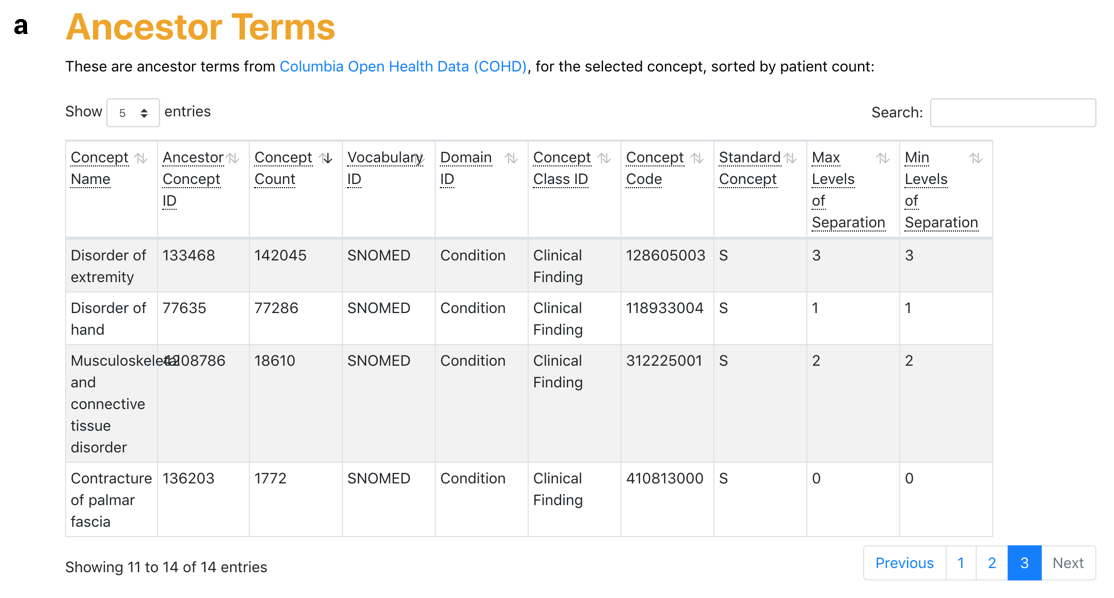

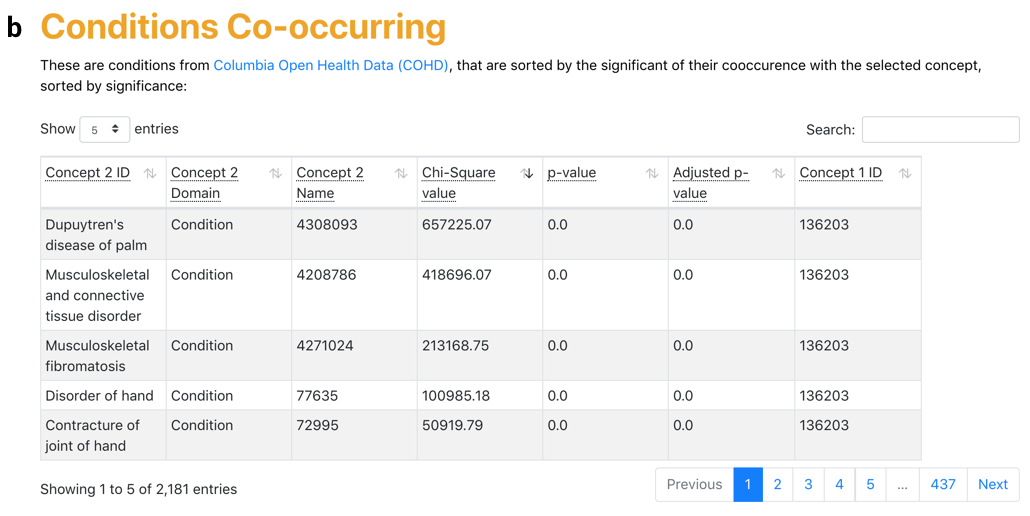

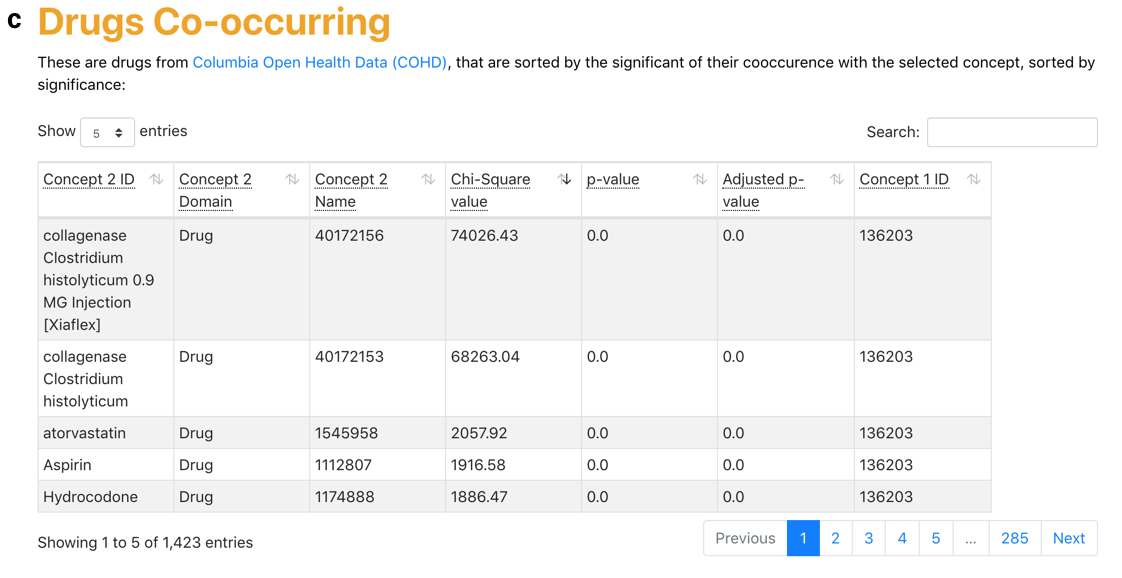

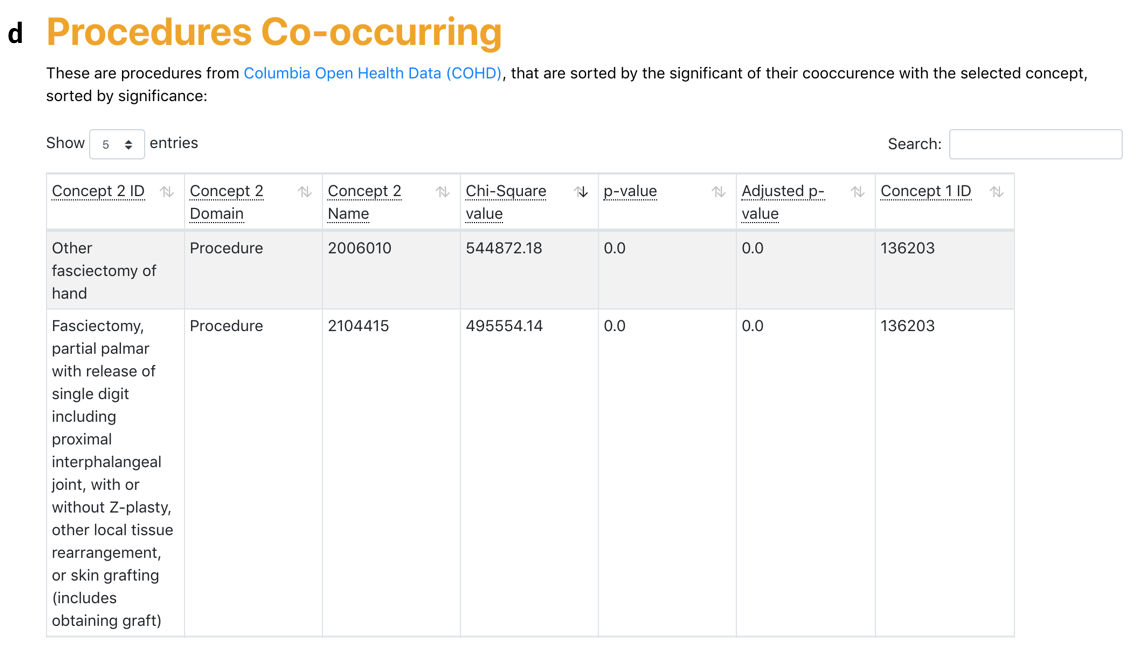


**Figure S4. Exploring the COHD page for “contracture of palmar fascia.”**

The results of all of this data is sorted by chi-square value, which is calculated based on the number of patients that do and do not have the first and second co-occurring terms in their notes to create a 2x2 contingency table.

**Disease synonyms, Gene Ontology data and more from Pharos**

Access to the disease portion of the Pharos [20] resource is provided in the Related Terms section. We provide access to related information to the search term: linked ontology disease names from Pharos (**Figure S5a**), drug target development levels for the drugs (**Figure S5b**), the protein/drug target families (**Figure S5c**), Reactome biological function pathways (**Figure S5d**), Gene Ontology processes, functions, and components (**Figure S5e-g**), linked UniProt disease names and tissue expression types enriched in targets linked to the UniProt disease names (**Figure S5h,i**).


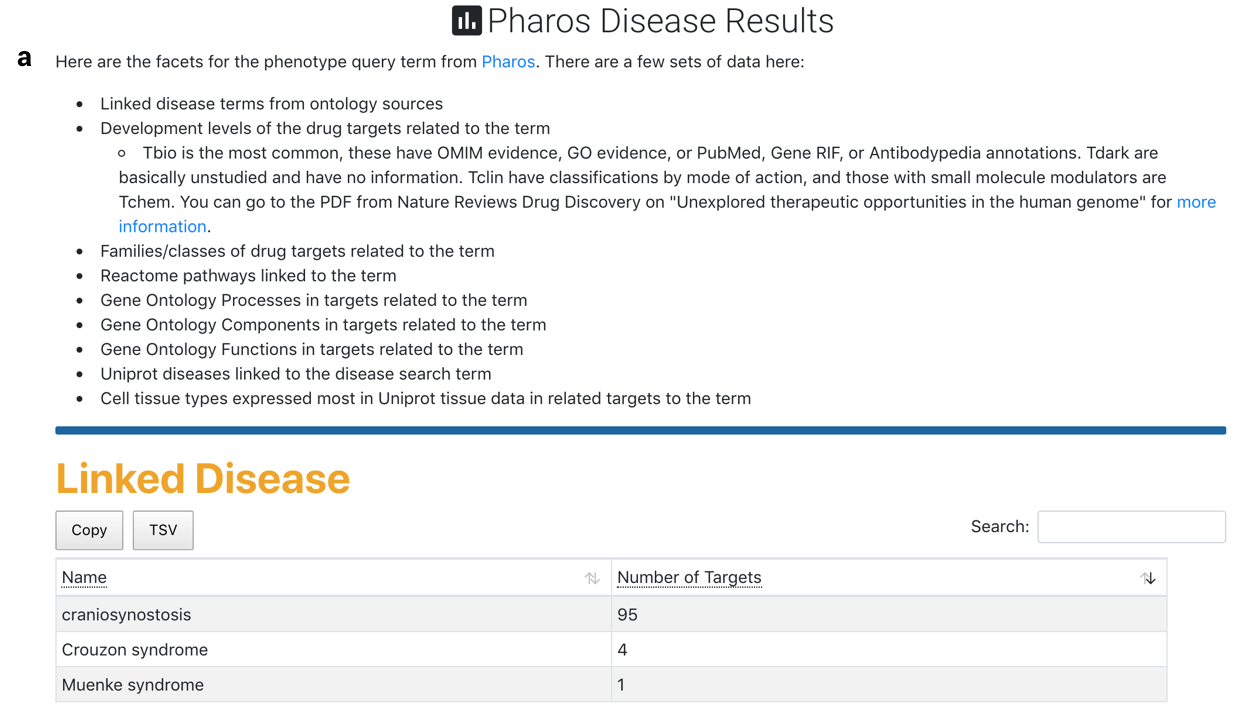


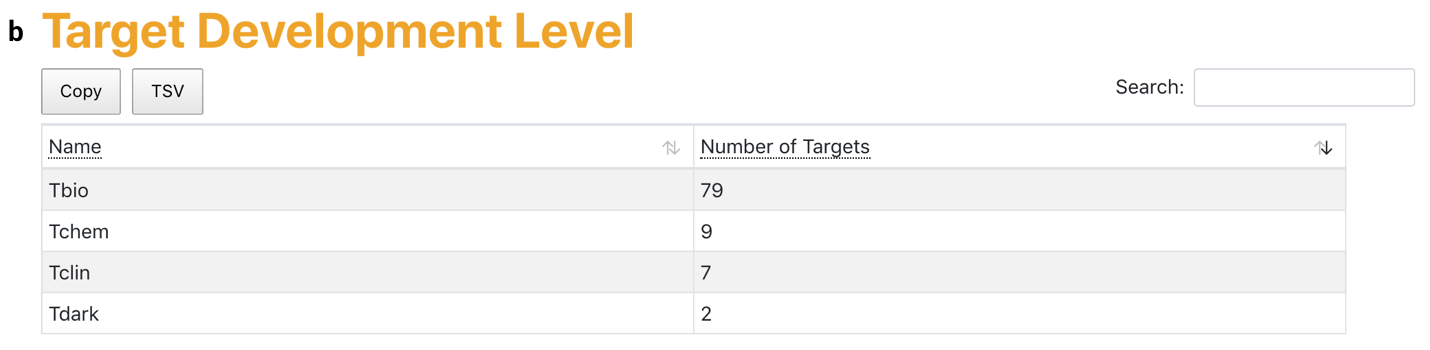


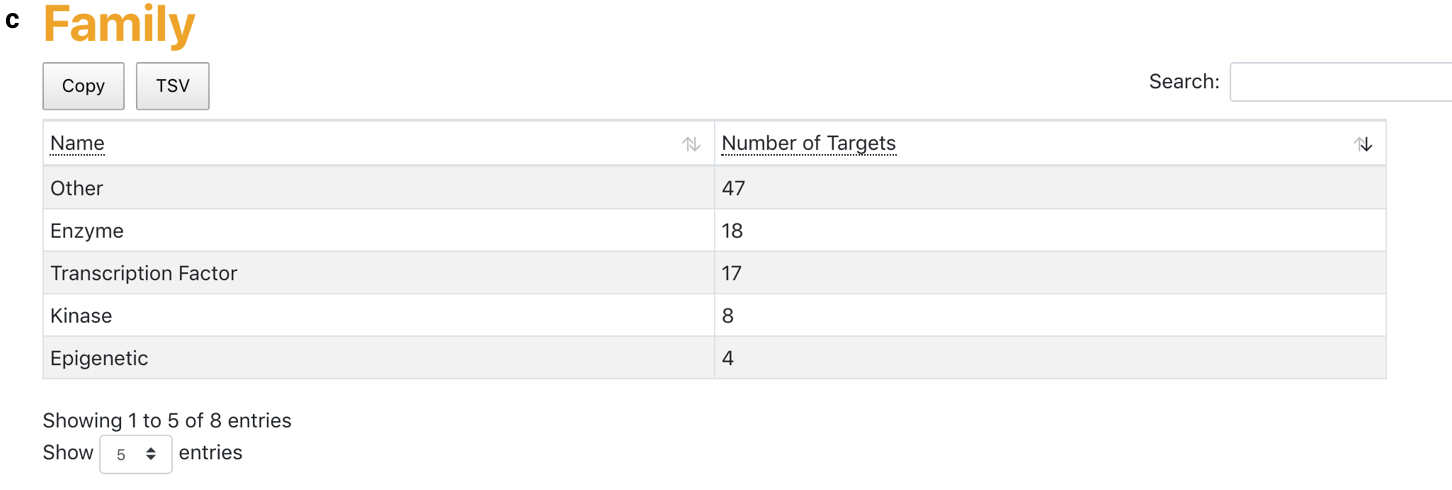

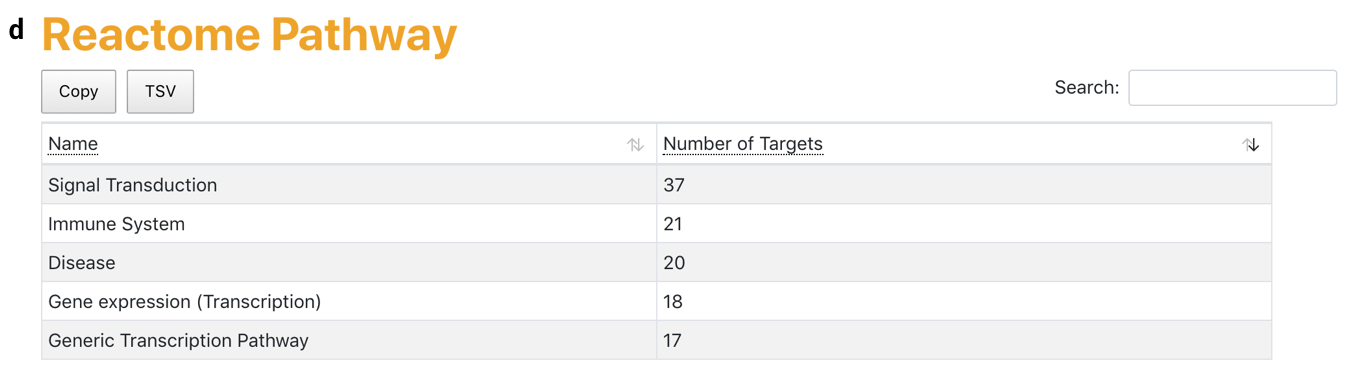

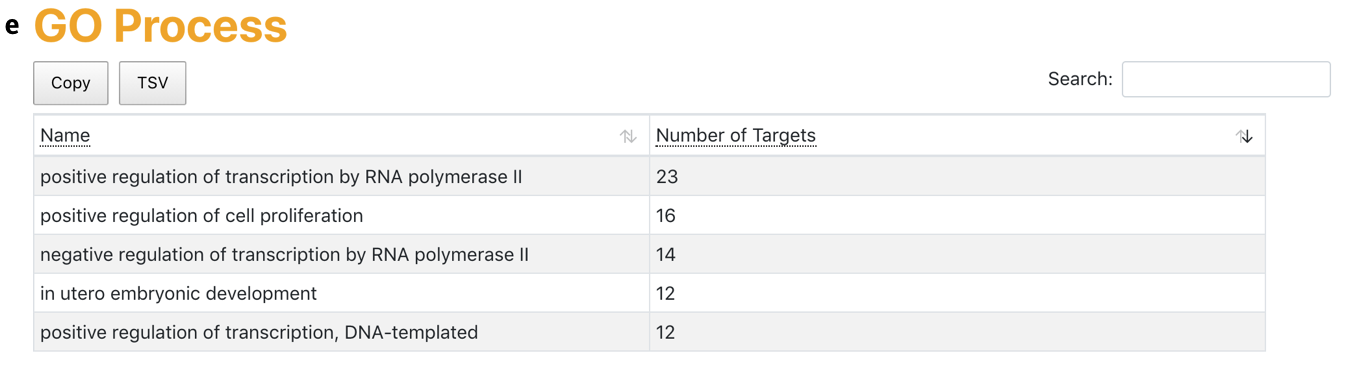

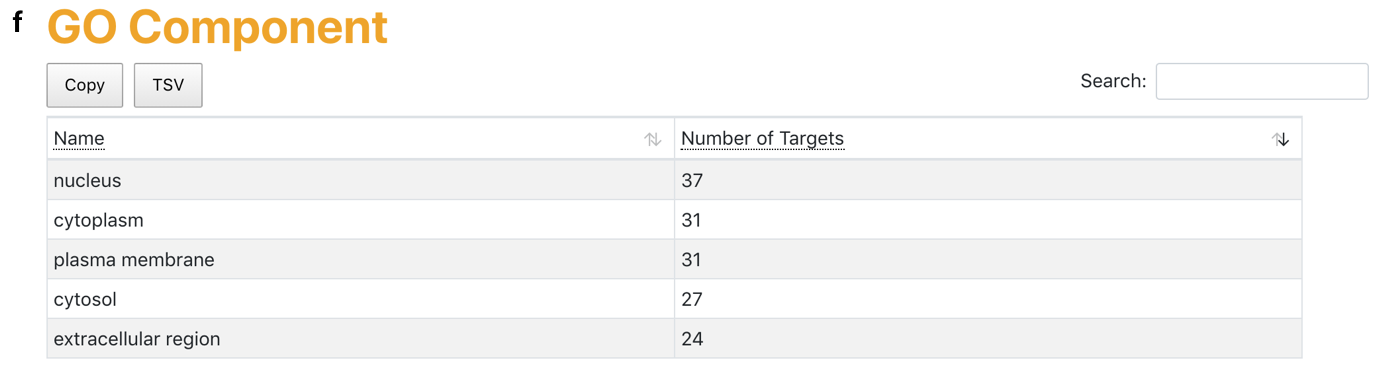

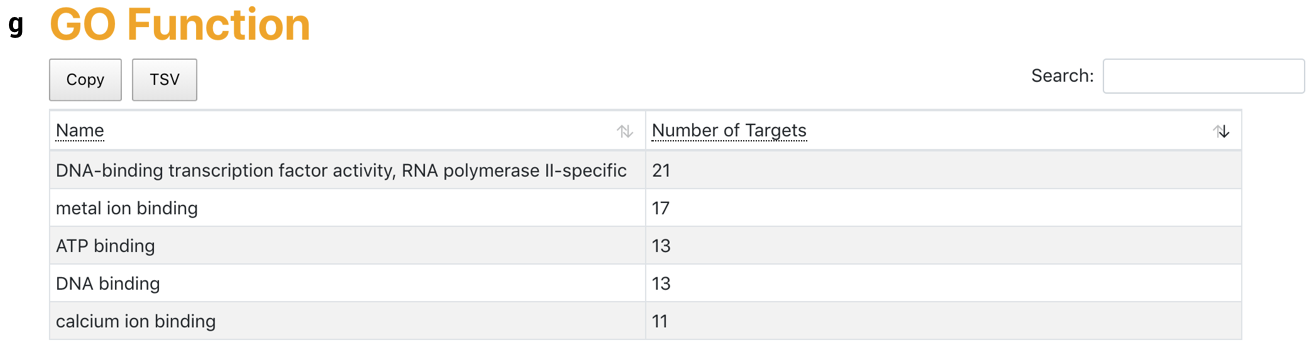

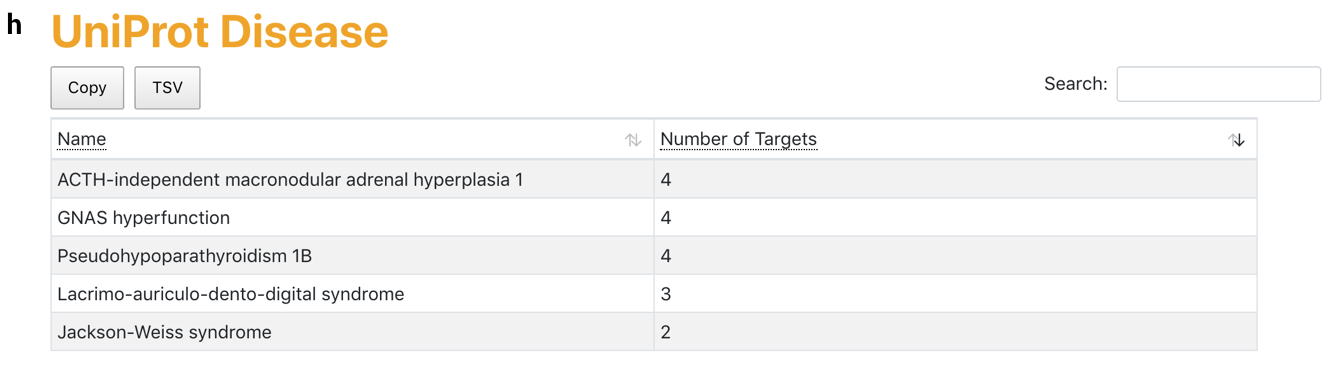

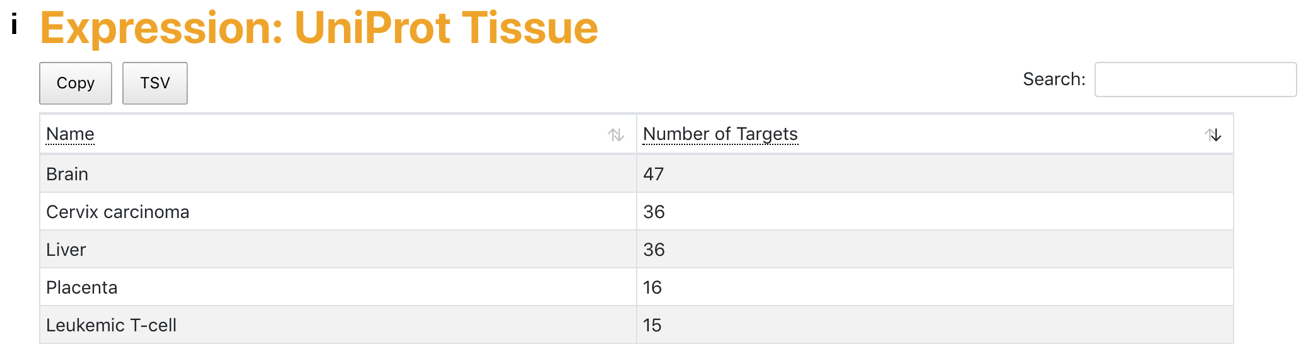


**Figure S5. Exploring the Pharos disease results page for “craniosynostosis.”**

**Disease terms related to phenotype term query**

If the user decides to type in a disease term instead of a phenotype term or the phenotype term is within the disease name itself, the ICD-10, OHDSI, MeSH, UMLS and DO results (**Figure S6a**) will display disease name aliases. However, if the user inputs a phenotype term and wants disease terms linked to that term which do not contain the query term itself, the HPO-linked disease data can provide this information (**Figure S6b**). If using the search term “palmar creases”, disease names that contain “palmar” or “creases” get boosted more if the linked HPO-term also contains these words, but disease names without those words also appear in the top five on the list such as “Primary hypergonadotropic hypogonadism-partial alopecia syndrome” from Orphanet.


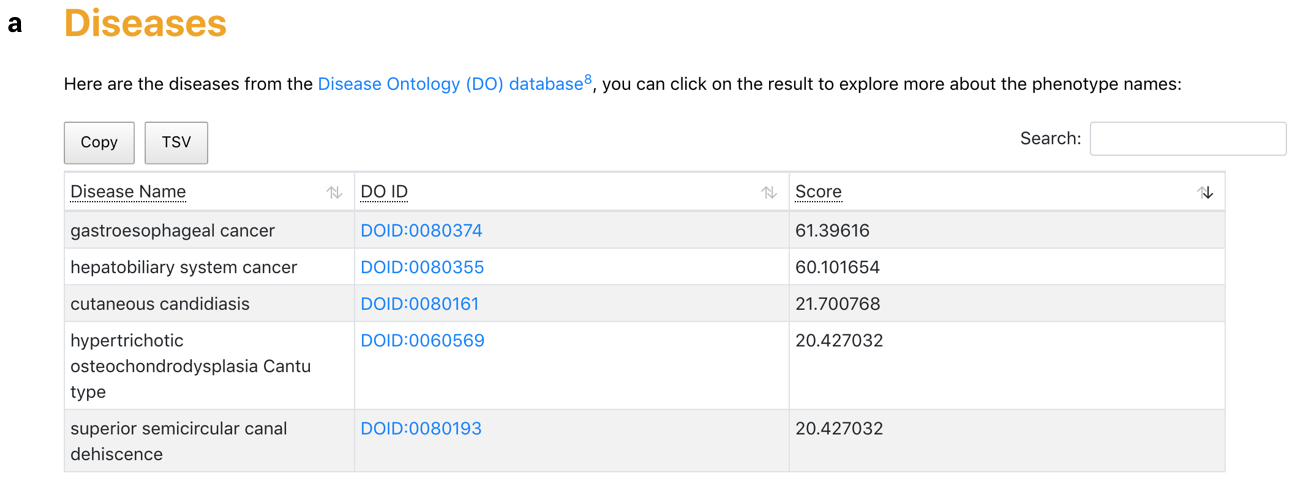

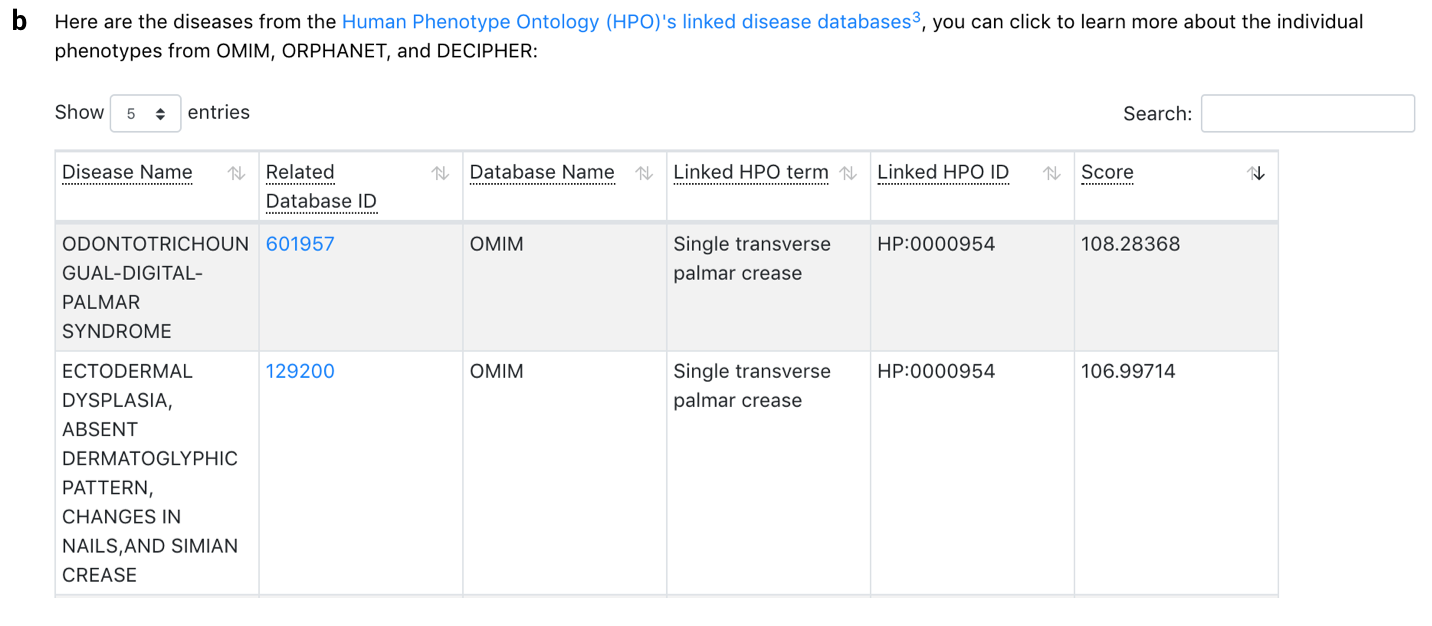
**Figure S6. Exploring the DOID and HPO-linked disease results for “palmar creases.”**

**Using Phen2Gene and Pharos to explore related genes**

Our tool, Phen2Gene, uses HPO terms to prioritize candidate genes for a potentially rare patient phenotype. For each HPO term, Phen2Gene ranks all of the genes based on ClinVar, OMIM, GeneReviews and Orphanet disease information and then further expands the gene list and re-ranks the genes based on gene–gene databases: HPRD, NCBI’s Biosystems Database, HGNC Gene Family and HTRI. It then aggregates all of this information for each HPO term, when used for more than one HPO term, and uses that to weight each gene collectively.

If taken from the “Phenotype search” page, the top HPO term result is automatically used to query Phen2Gene, and if from the “Patient notes” page, all HPO terms extracted are used for the Phen2Gene query. Phen2Gene returns the top 1000 gene results sorted by rank. The user can click the gene name links to go to MedlinePlus [21] (**Figure S7a**). The user can obtain potential drug targets from Pharos as well, and these link out to the PhenCards Pharos drug target results page if clicking the symbol, and the official Pharos site page if clicking the UniProt ID (**Figure S7b**).


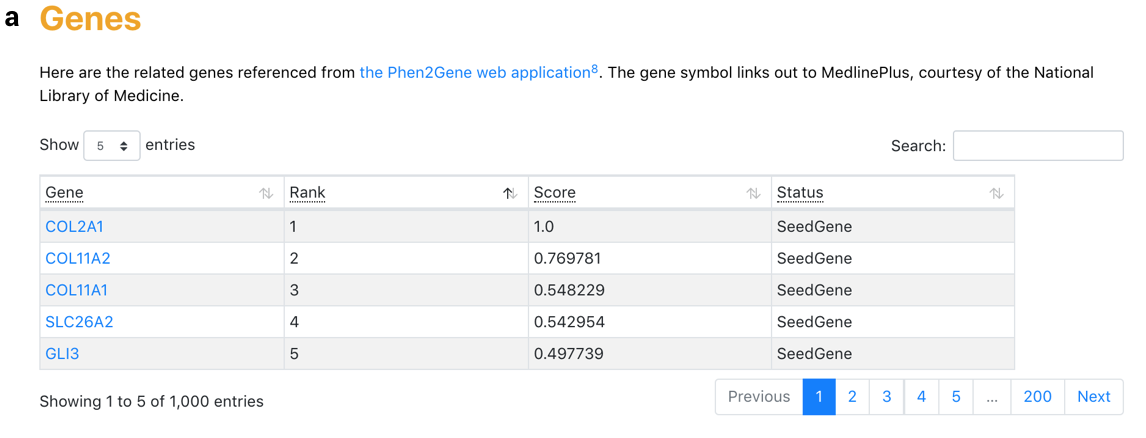

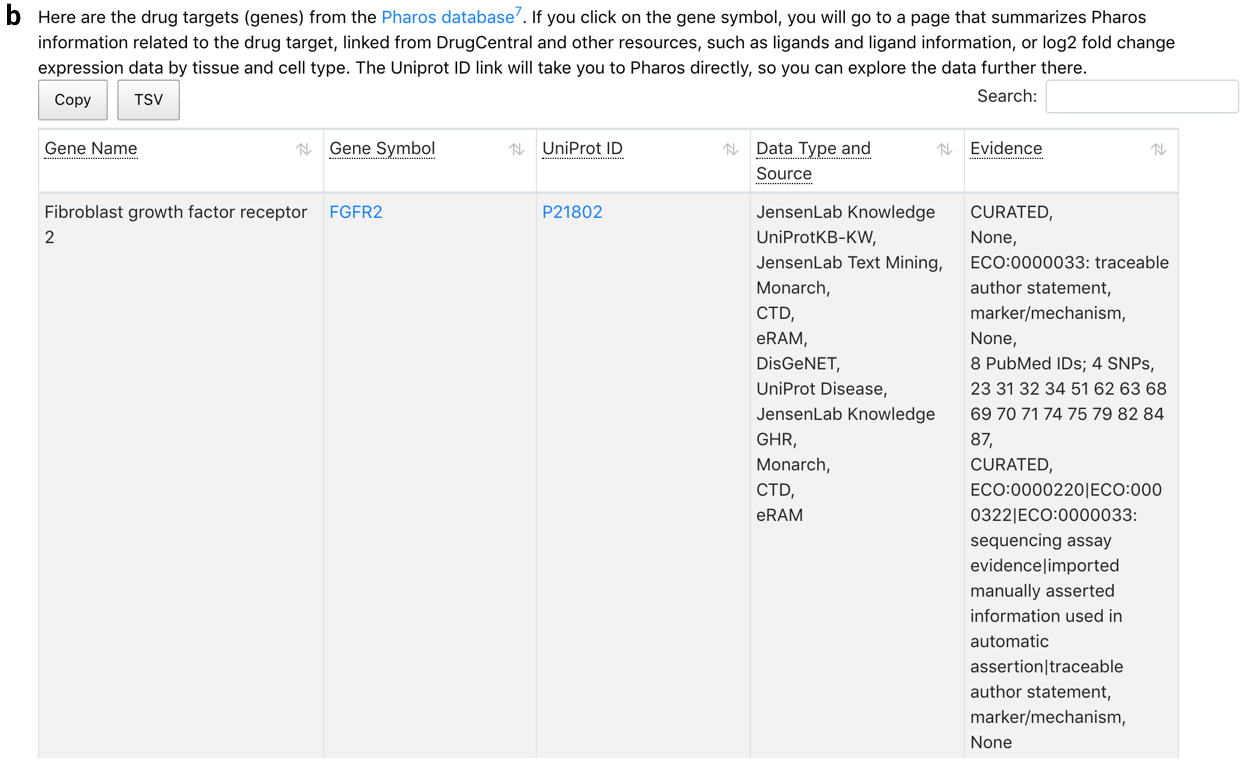
**Figure S7. Phen2Gene and Pharos provide potential gene targets. a.** Phen2Gene results for the HPO term “cleft palate”. **b.** Pharos results for the term “craniosynostosis”.

**Pharos provides detailed drug target information**

PhenCards parses Pharos data on drug targets on the drug target results page. The user can learn how novel of a target the gene is based on literature citations, its development stage in the DrugCentral database, and protein family (**Figure S8a**). The drug target development levels can vary: Tbio is the most common, these have OMIM evidence, GO evidence, or PubMed, Gene RIF, or Antibodypedia annotations. Tdark drug targets are basically unstudied and have no information. Tclin targets have classifications by mode of action, and those with small molecule modulators are Tchem. The user can also discover potential drugs that bind to the target and if they are published or already drugs (**Figure S8b**). The expression of the target in different tissue types in the various expression databases in Pharos can be found as well (**Figure S8c**). Finally, the user can obtain protein-protein interaction data for other proteins with the target and likelihood scores for their interaction with p-values based on interaction scores calculated using information from their respective databases (**Figure S8d**). pNI is the probability the interaction is non-specific, pInt is the probability the interaction is true, and pWrong is the probability the interaction is incorrect. Here are the target details for the drug target FGFR2 from Pharos. There are a few sets of data here:

**
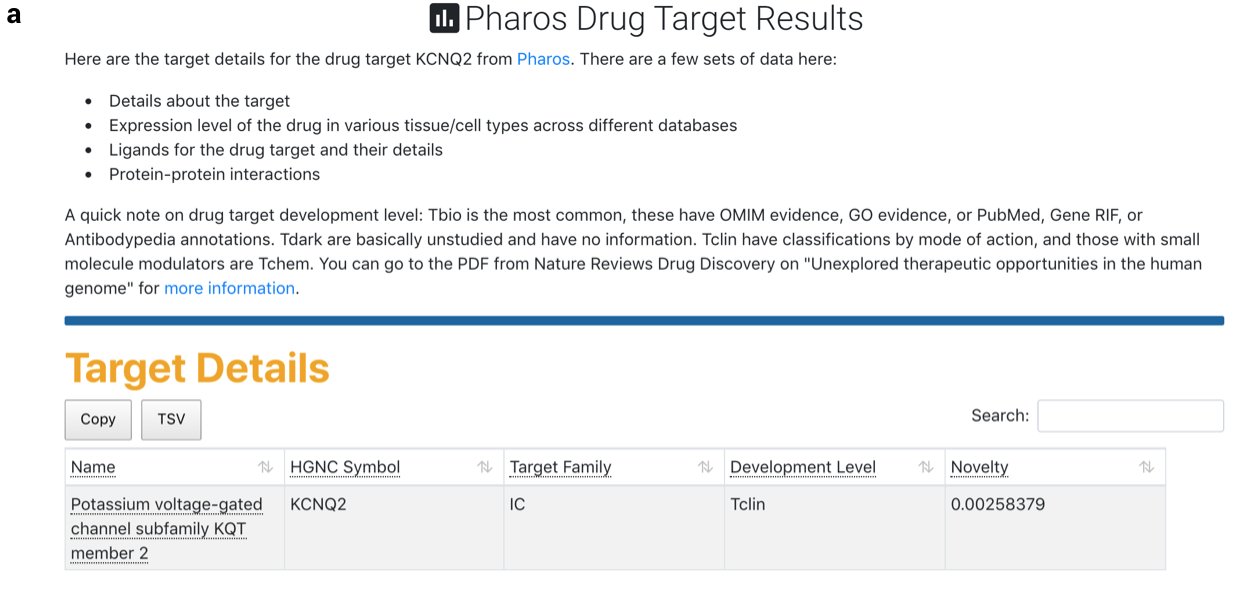

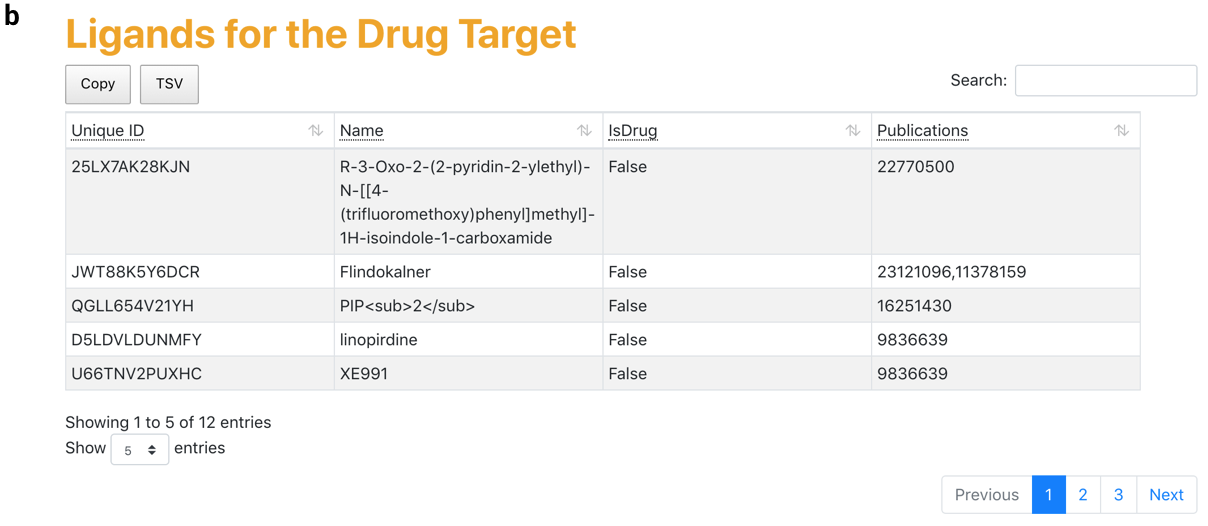

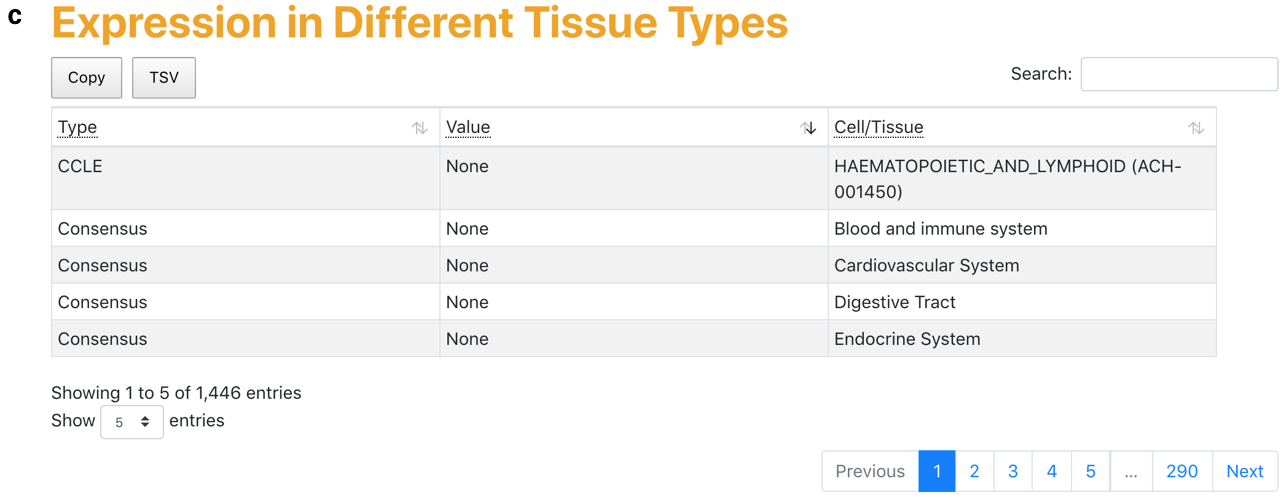

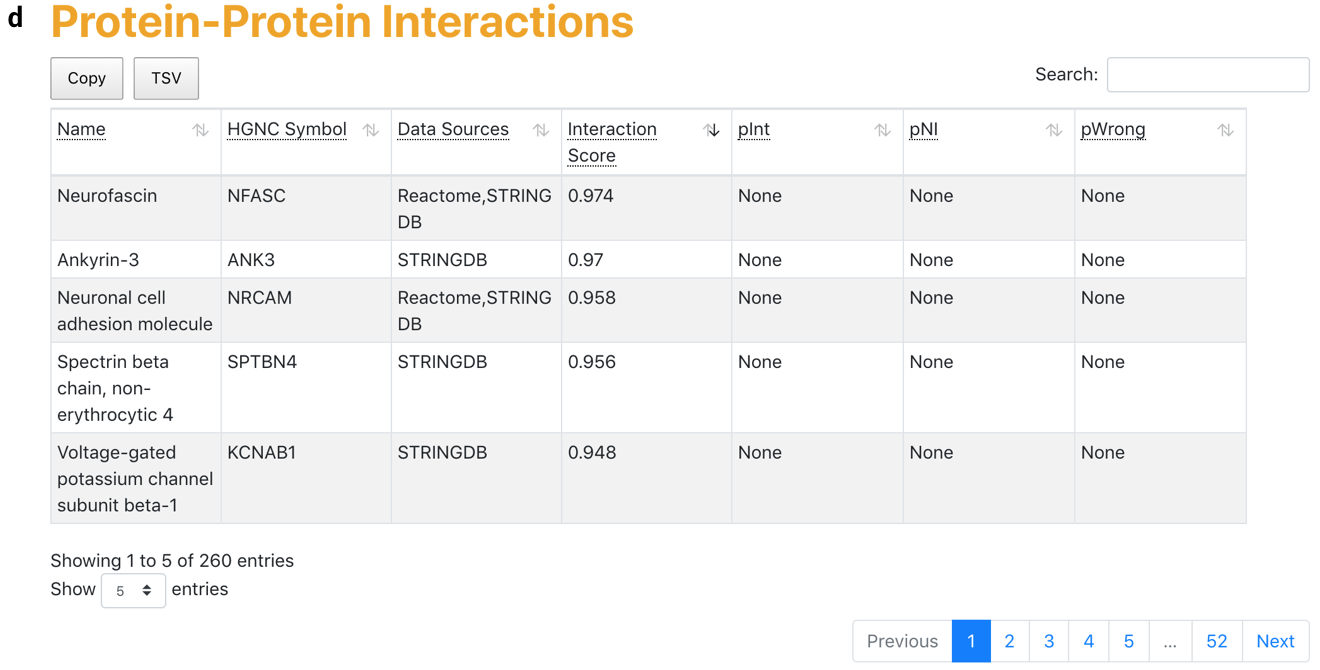
**

**Figure S8. Pharos provides various gene target information. a.** Details of the target, development, family, and novelty. **b.** Potential drug ligands for the target. **c.** Expression of the target in different tissue types and datasets. **d.** Protein-protein interactions for the target.

**Pathways linked to phenotype terms and related disease**

If on the “Phenotype search” results page, the user can click the links out to see KEGG disease and linked pathway results for the search term (**Figure S9a**) as well as the more expansive pathway results from Pathway Commons and the disease and biological ancestral pathways linked to the search term (**Figure S9b**). This is another useful avenue of disease exploration, as the first Pathway Commons results “Peters-plus syndrome” is a rare disease with cleft palate as a symptom that is not seen in the top hits for linked disease terms on the patient page. Thus, not only is this a way to click the pathway links and learn more about the mechanisms of the phenotype on Reactome and other resources, but also to discover other potentially related diseases.

**
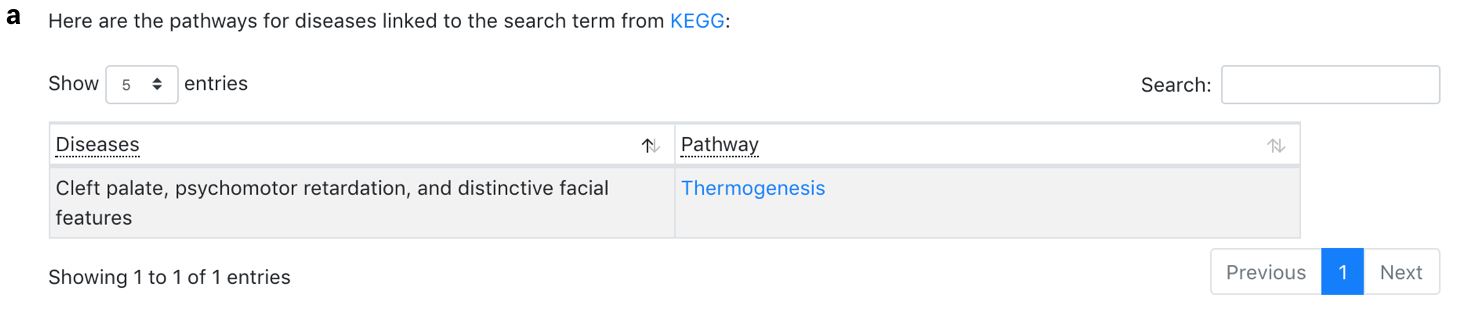

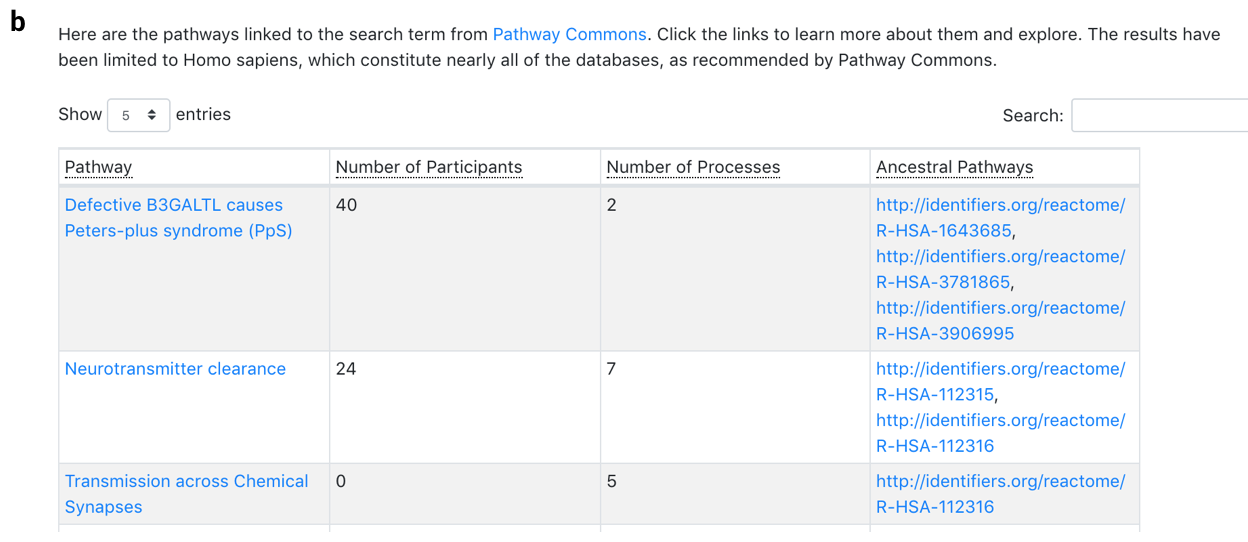
**

**Figure S9. Pathway results for the search term “cleft palate.”**

**Exploring drug and clinical trial data related to phenotype**

The phenotype term query can also be used to search several drug databases, including DrugCentral[22] and the company websites for APExBio and Tocris drug products. But the primary drug search capability comes from the openFDA and ClinicalTrials.gov resources. In the openFDA page, one can determine if the phenotype term is an adverse reaction in the FAERS database, if so, what drugs cause it, what age groups typically experience the reaction, what methods of administration were used, the patient weights, the outcome of the reaction (fatal or otherwise) and any drugs that caused the reaction. Additionally, the page will search from the perspective that the search term is the condition itself, and return drugs typically prescribed for that condition, reactions to the drugs prescribed for that condition, and forms of administration of those drugs. A multitude of investigation can be done with this amount of data.

We demonstrate a test of DrugCentral using the term “cancer” and this shows likelihood calculations for adverse drug events for all drugs (where the p-value is less than 0.05) that lead to conditions like the search term, which are calculated using openFDA FAERS data split by male and female (**Figure S10a,b**), on the DrugCentral backend. Additionally we provide drug indication, off-target, and contraindication data similar to the search term using OMOP data from DrugCentral (**Figure S10c**).


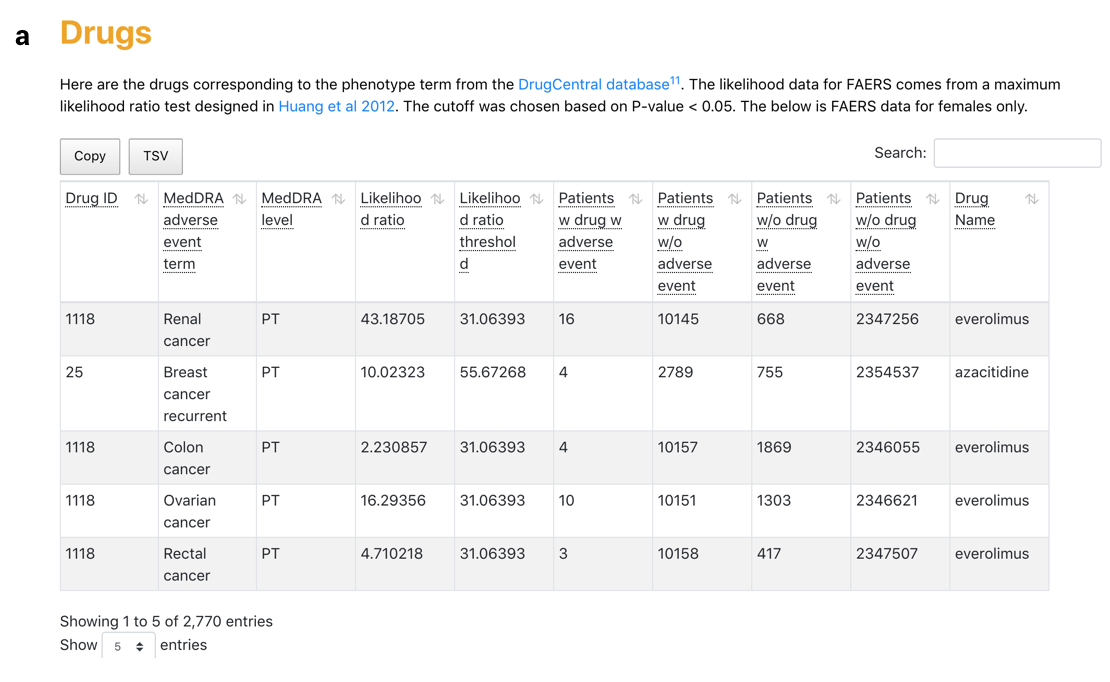

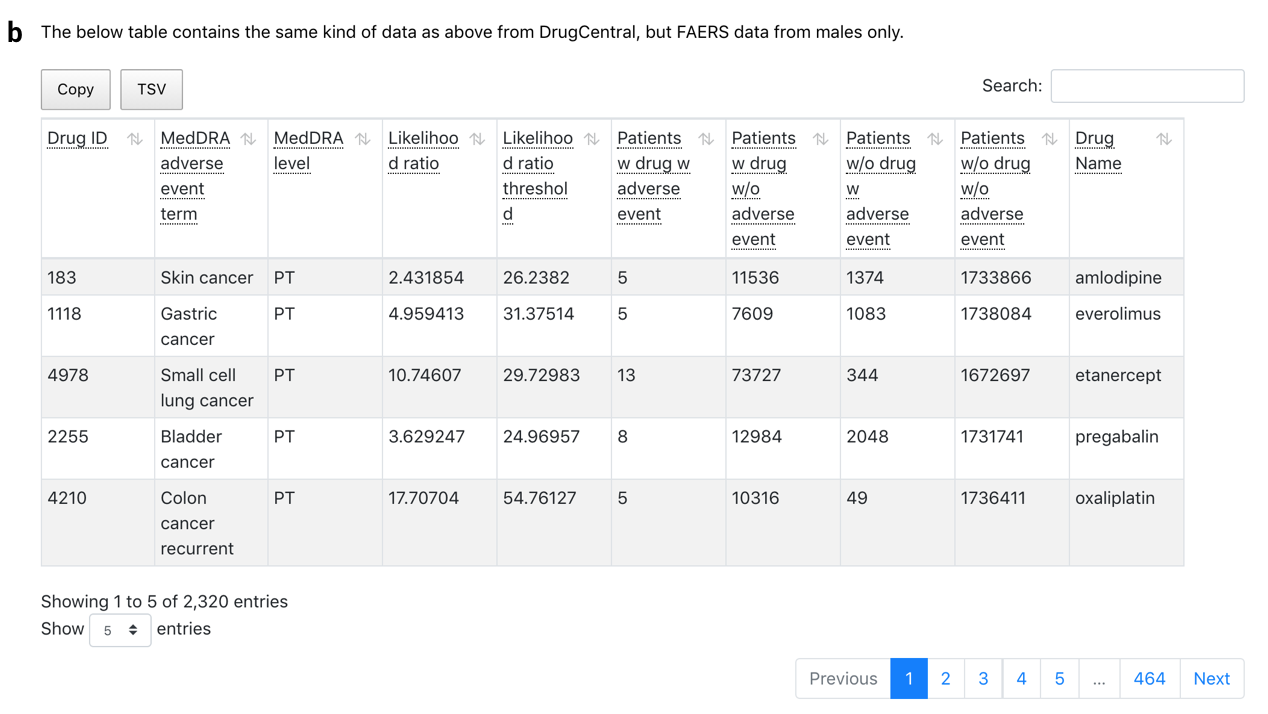

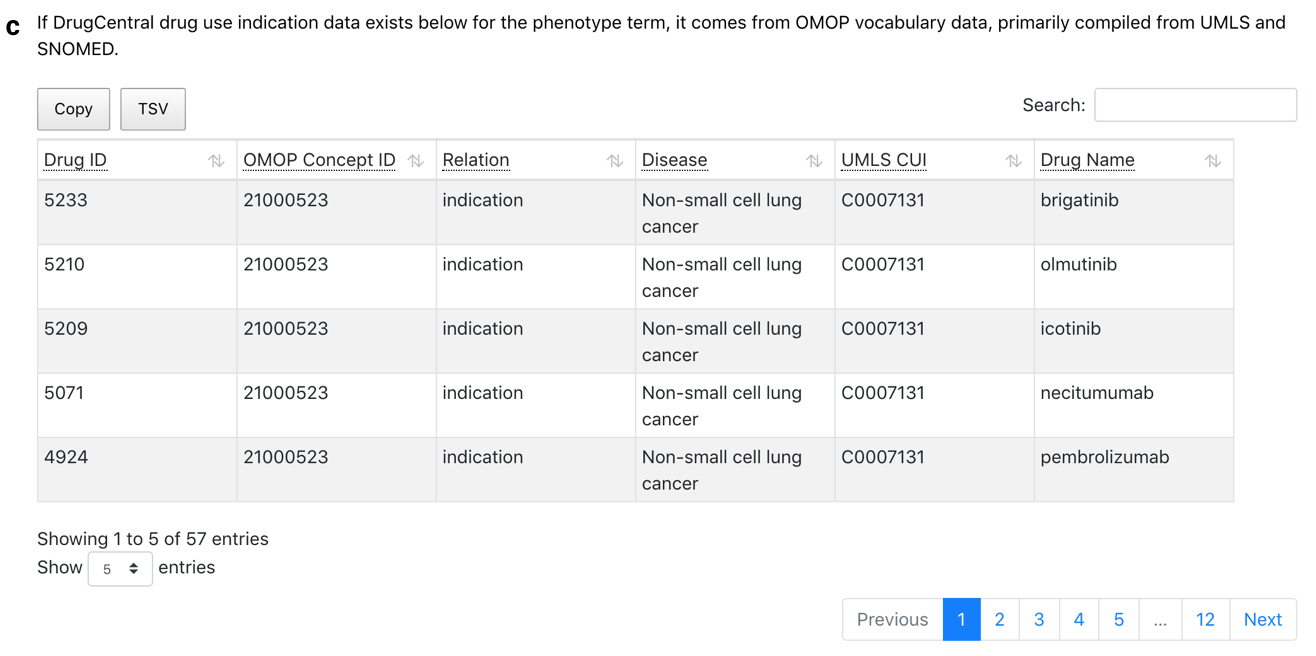


**Figure S10. DrugCentral results for the search term “cancer.”**

A quick test case with “cleft palate” shows that patients who have this reaction tend to have it during birth, caused when a mother has been prescribed lamotrigine during pregnancy for her seizures[23] (**Figure S11a,b**). In addition, a search through the Clinical Trials section of the site reveals several methods to deal with cleft palate and its complications: surgical repair, antibiotic prophylaxis during surgery, and even an educational study on the subject (**Figure S11c**).


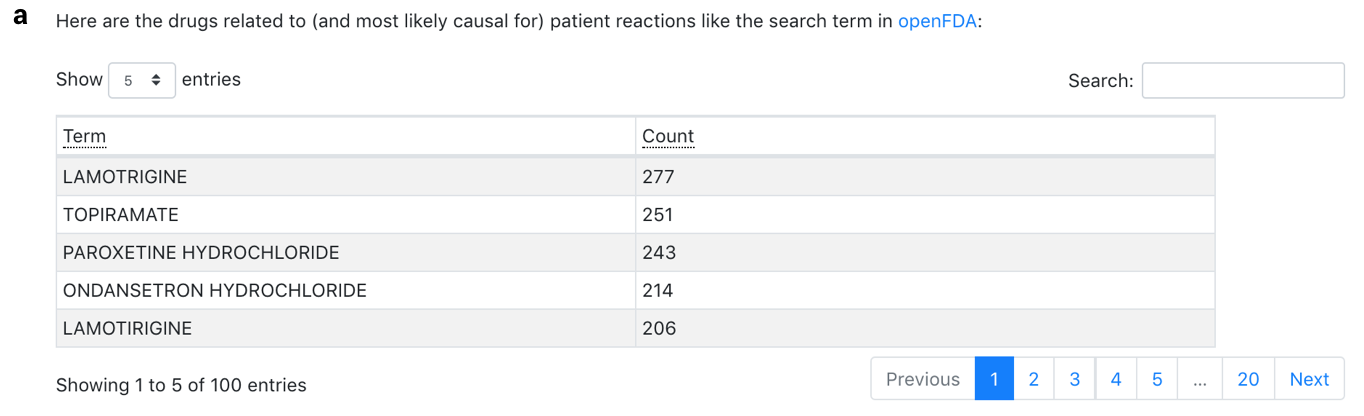


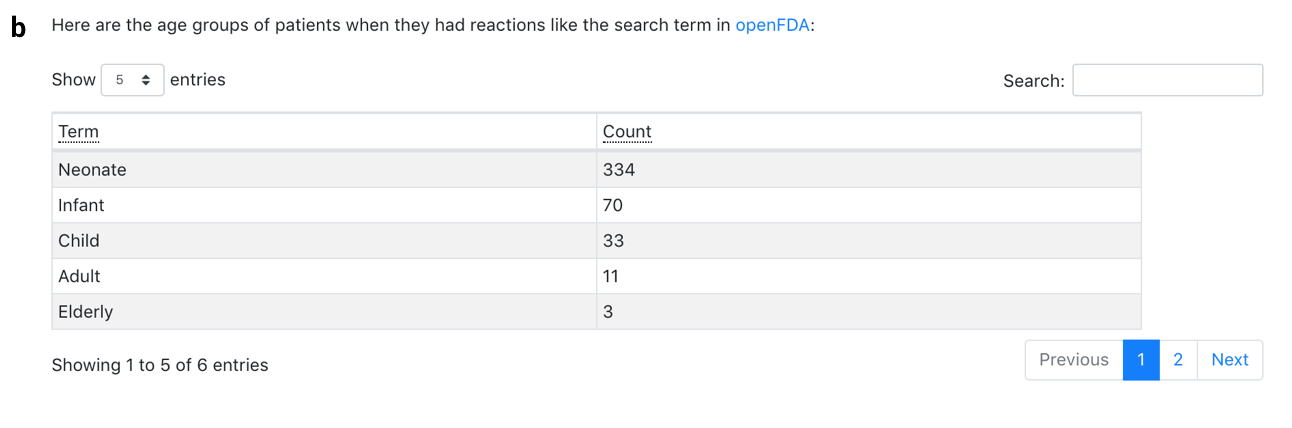

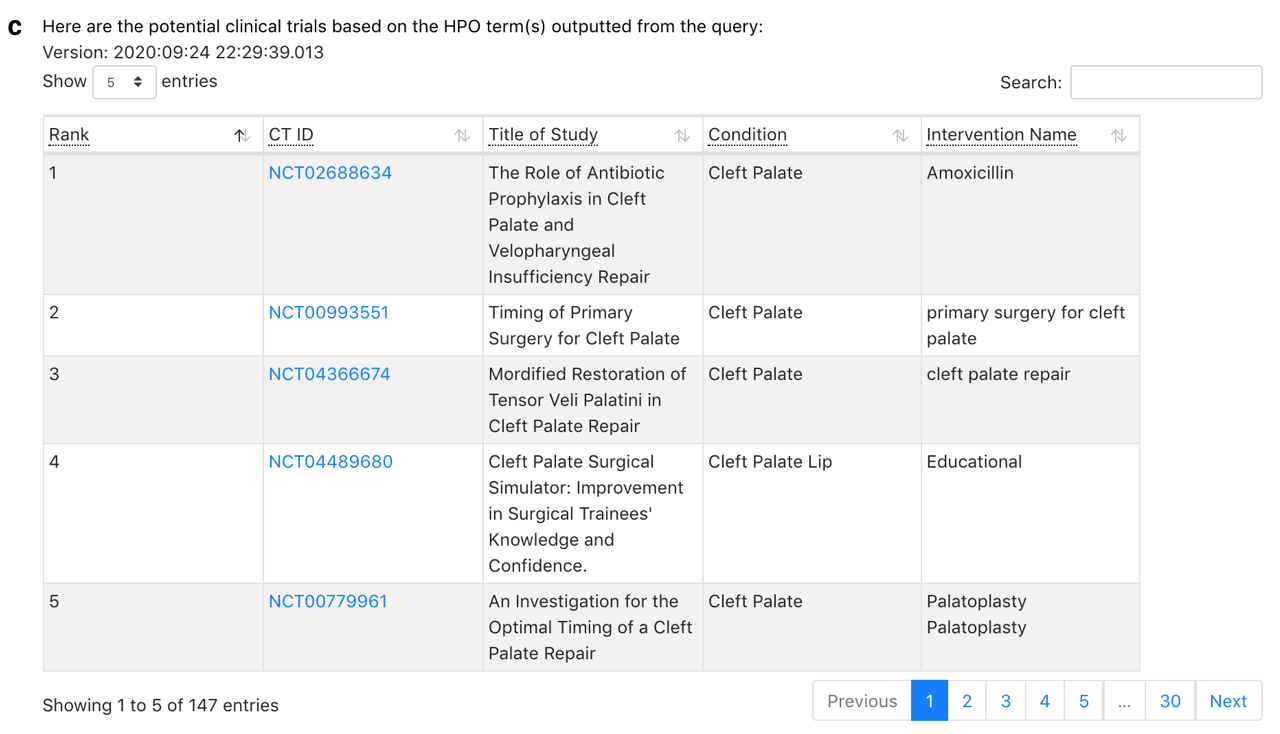


**Figure S11. Drug and clinical trial investigational results for the search term “cleft palate.”**

**Funding support, treatment, and collaborative information**

Using “cancer” as our search term, we can see many nonprofit foundations have been created to help patients and provide grants for cancer research (**Figure S12a-c**). This data comes from Open990 and the Internal Revenue Service, and is freely accessible under public domain. We hope this will assist researchers in finding non-governmental collaborators, assistance, and sources of funding.

The IRS nonprofit data is hosted freely on AWS, and the URL links navigate to XML files with a myriad of information about the company, such as who runs it, money going in and out of the company and contact information. Open990 parses some of this data from these XML files and summarizes it, though not always accurately.

**
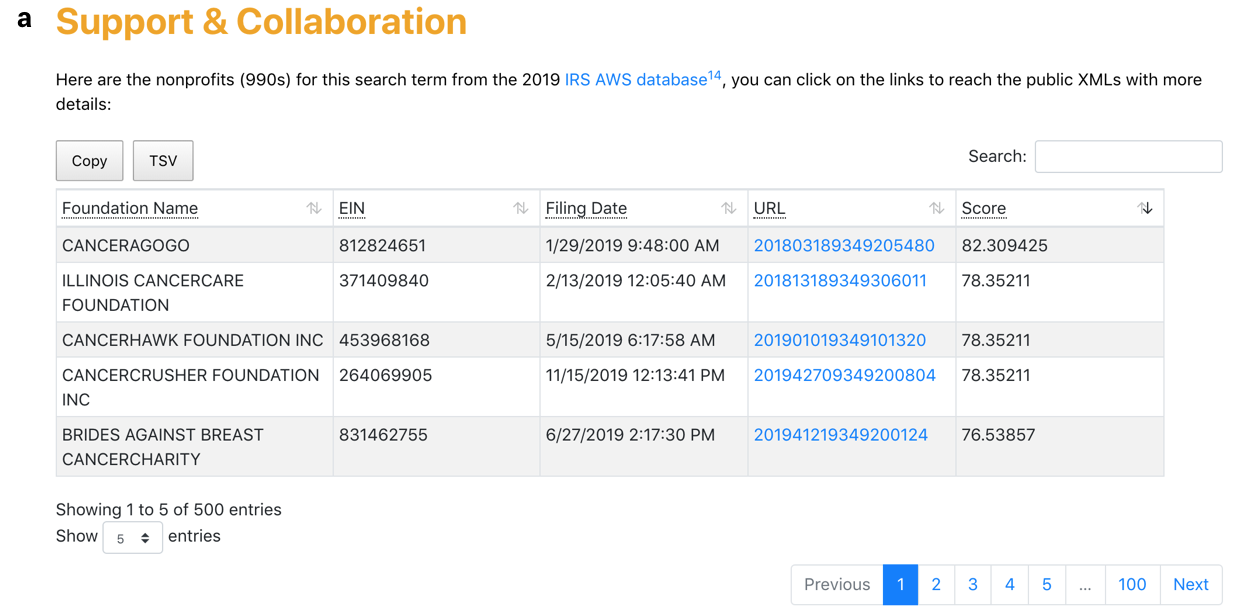

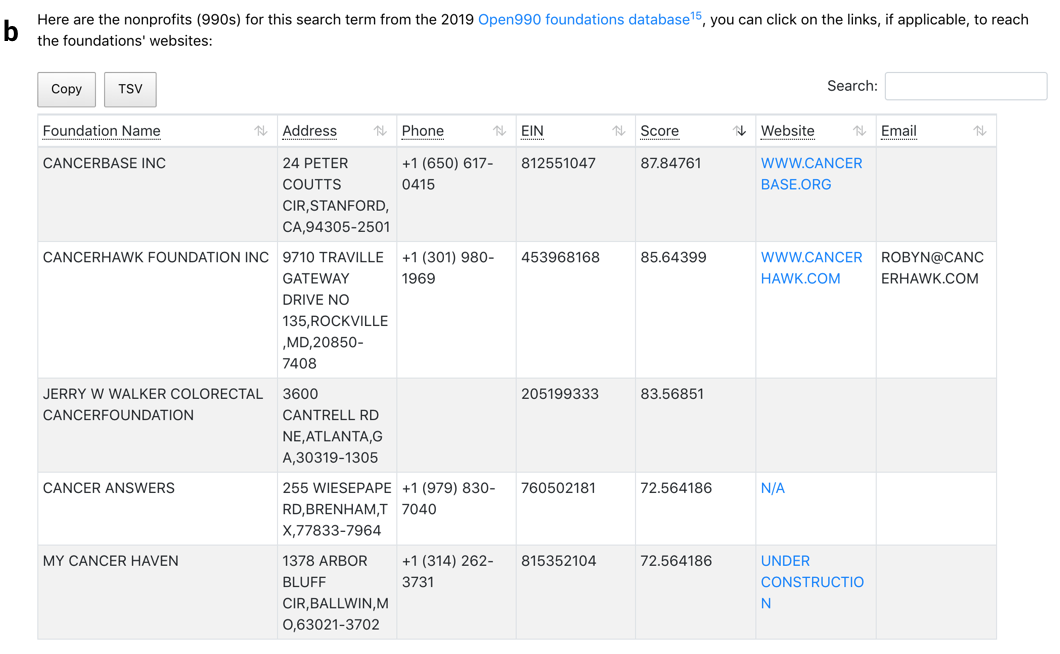

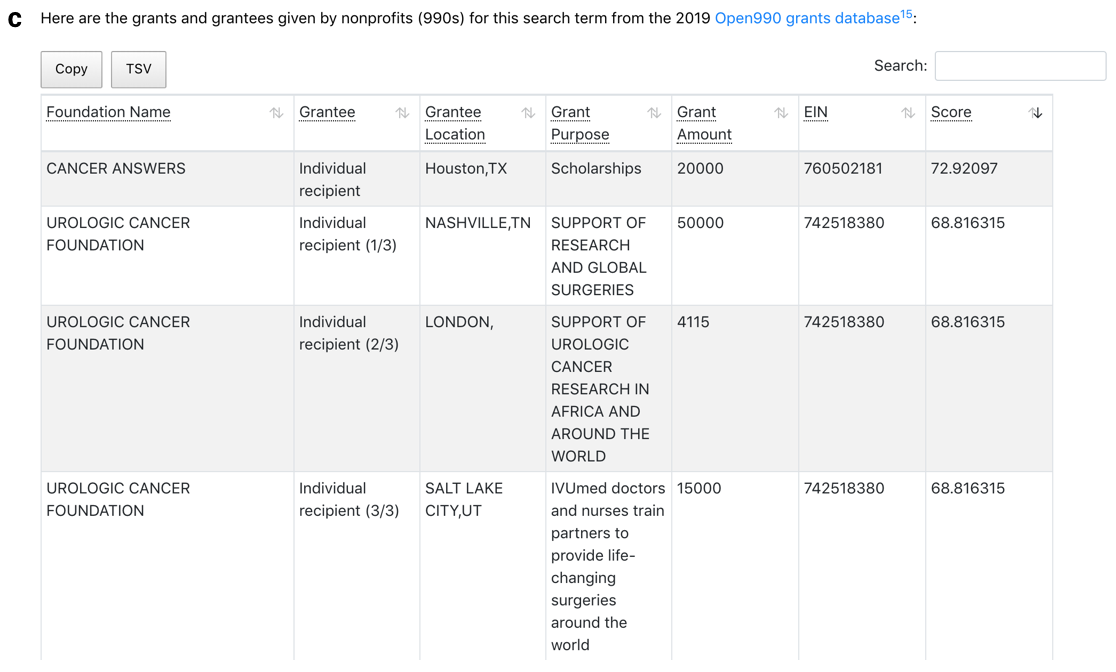
**

**Figure S12. IRS and Open990 foundation and grant data for the search term “cancer.”**

We also provide sources of government-funded grant data. Using “craniosynostosis” as our search term, we can find active NIH funding opportunity announcements (FOAs) from which scientists can attain grant money (**Figure S13a**). PhenCards can find active government projects from the NIH, NSF and other government agencies. This may help provide the user with potential collaborators or physicians who could assist a patient (**Figure S13b**). Lastly, we link to Direct2Experts which finds participating institutions’ physicians and researchers that may be able to provide medical or surgical treatment for a condition, as well as collaborate with the potential expert user. These are experts in several medical institutions that are likely to specialize directly in the user’s phenotype. The user can click on the "Number of Experts" links to reach each respective academic medical institution’s results pages that are in this network. This will help the user find MDs, PhDs, genetic counselors and others specialized in your phenotypic search term (**Figure S13c**).

**
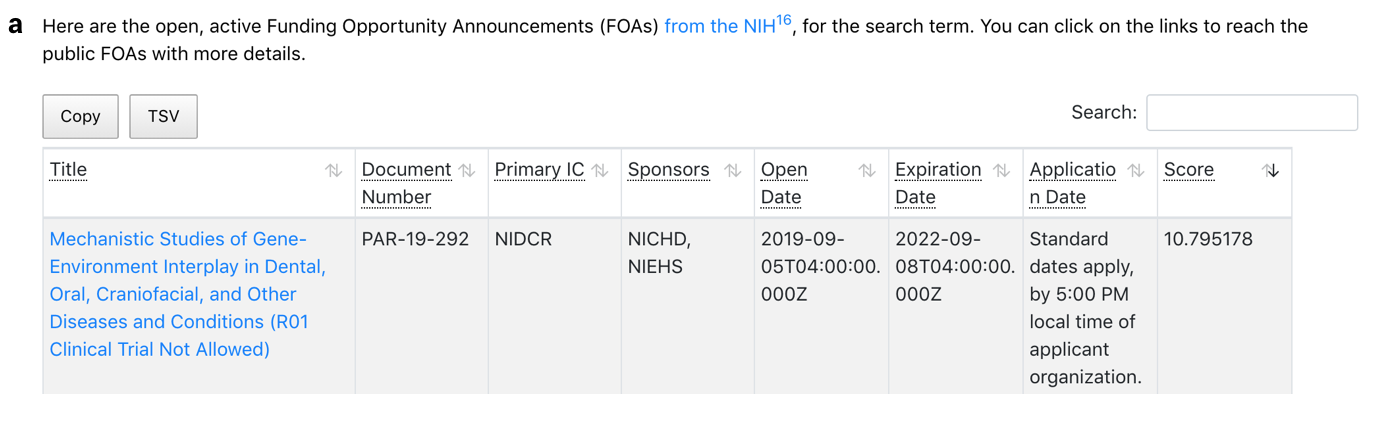

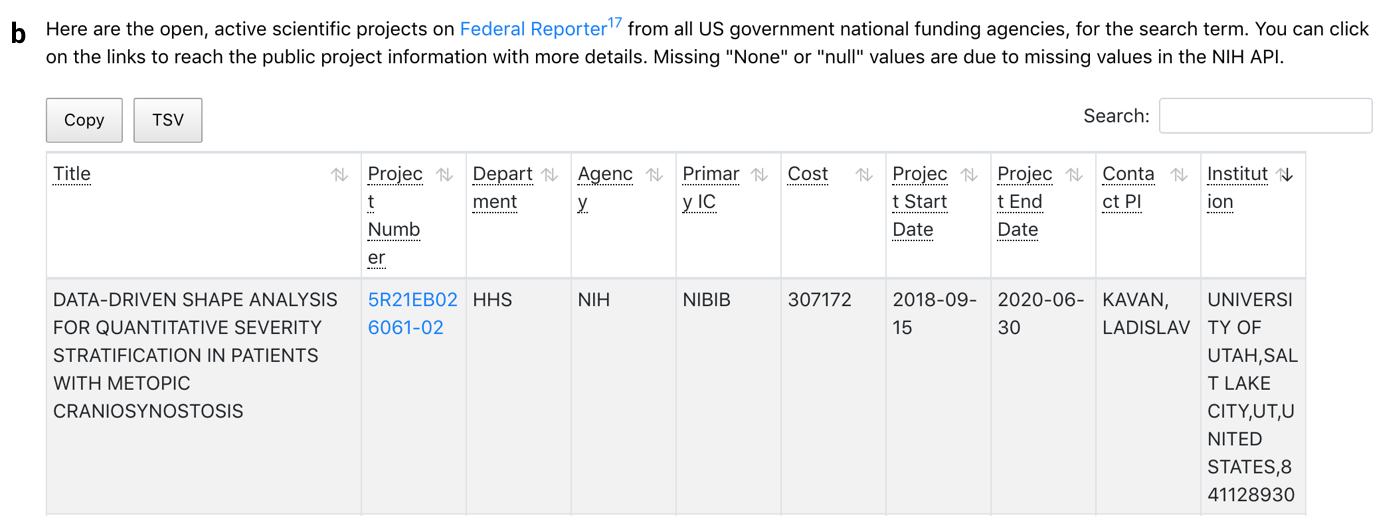

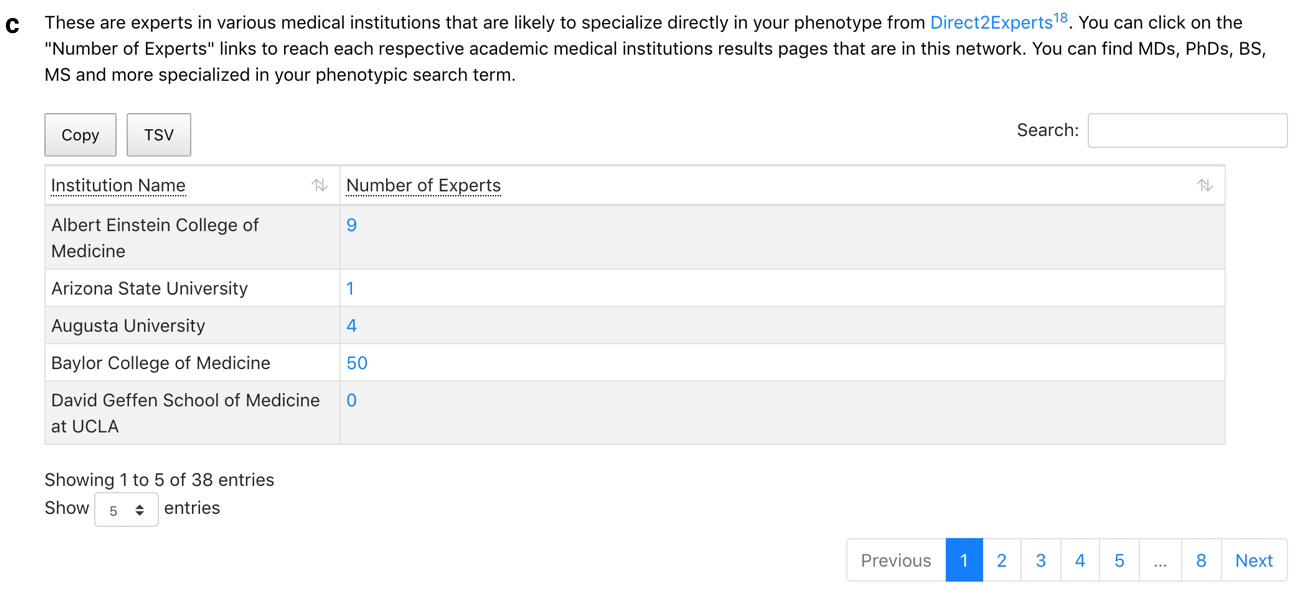
**

**Figure S13. NIH FOAs, Federal Reporter data for active projects and specialty physician and research collaborator data from Direct2Experts for the search term “craniosynostosis.”**

**Understanding the patient page**

After submitting clinical notes, the HPO terms are highlighted in green if used, and red if negated in Doc2HPO. The Phen2Gene results for the terms are displayed, and the gene names can be clicked on to search MedlinePlus. The terms are also used to predict the potential causal disease using Elasticsearch. The extracted terms are used to search ClinicalTrials.gov and can be individually clicked on to redirect the user to results from the “Phenotype search” tab (**Figure S14**). The extracted HPO terms also predict the most likely diseases and syndromes described by the patient notes (**Figure S15**). This disease prediction is done by exact (not fuzzy) matching each HPO term to its respective linked diseases in OMIM and Orphanet from the HPO database. Then we rank each disease by the combined elasticsearch score of those linked terms, which is based on Lucene index query matching. The higher the score, the higher the disease ranks. This score is based on the Okapi BM25 algorithm, where each query score is

$$score(D,Q)=\sum_{i=1}^{n} IDF(q_{i})*\frac{f(q_{i},D)*(k_{1}+1)}{f(q_{i},D)+k_{1}*(1-b+b*\frac{|D|}{avgdl})}$$

where D is the document and |D| is its length, avgdl is the average length of all documents, Q is the query containing keywords q1,…,qn, and k1 and b are arbitrary parameters where k1 is often either 1.2 or 2.0 and b is 0.75. IDF is inverse document frequency for each query keyword.

Finally, The PhenCards patient page provides a custom literature search that can be used easily with a keyboard or mouse to add or remove terms and make more or less specific by adding or removing “OR,” which ports the HPO terms to Google Scholar for investigation (**Figure S16**).

**
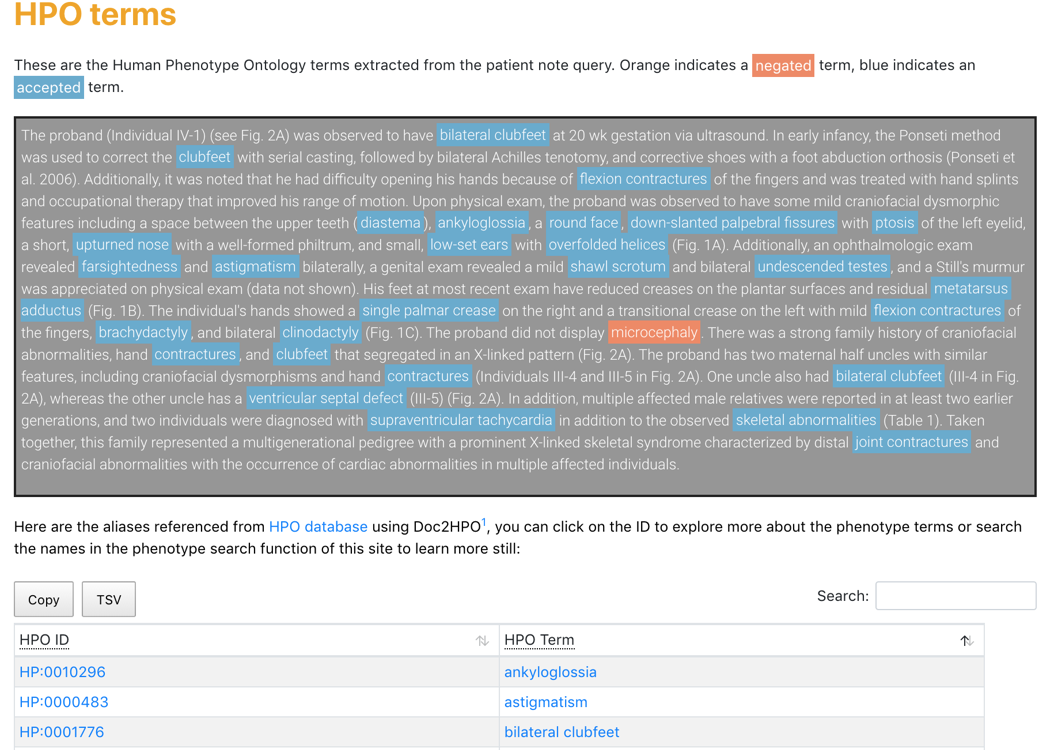
**

**Figure S14. Patient HPO term results for the sample notes on the PhenCards site.**

**
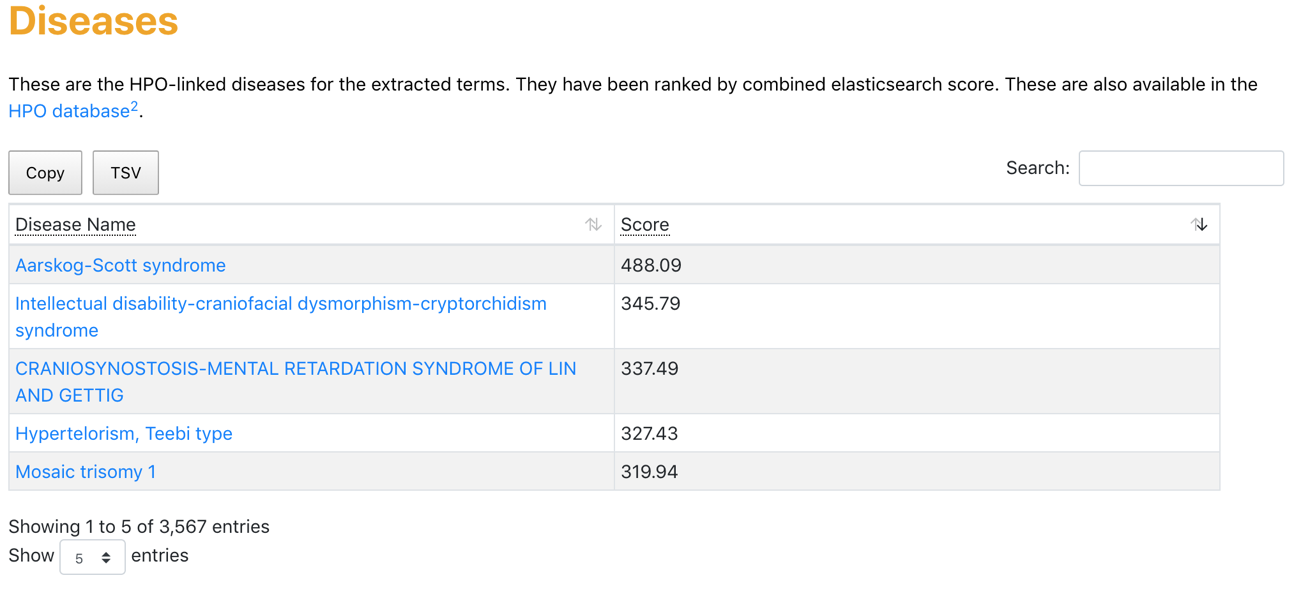
**

**Figure S15. Disease prediction rankings for the sample notes for the patient page of PhenCards.**

**
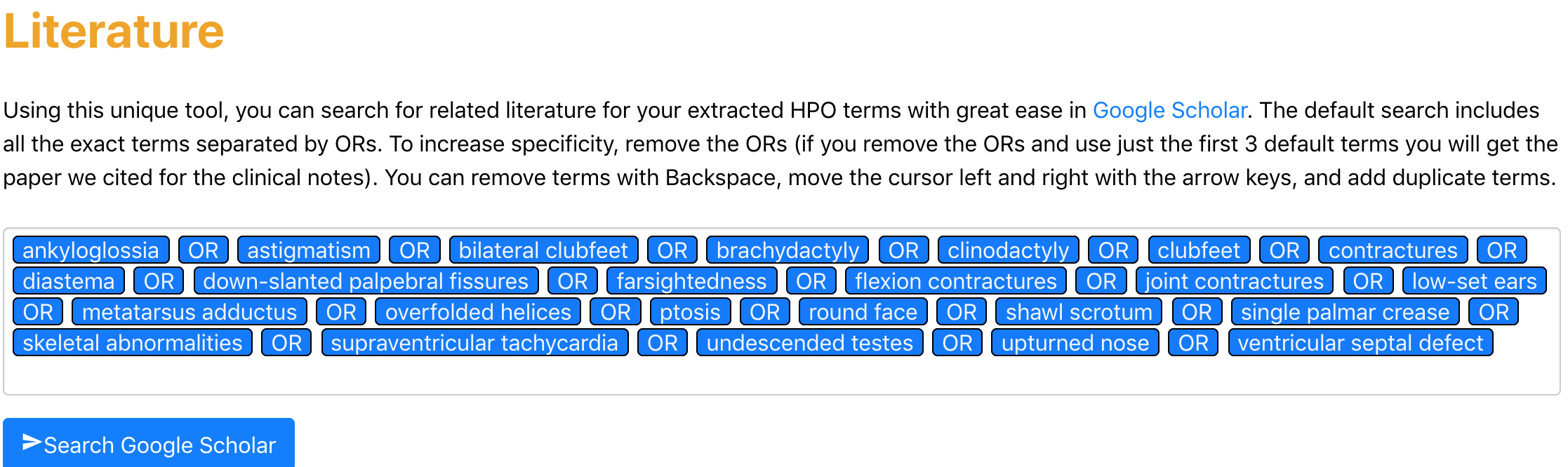
**

**Figure S16. Custom, editable Google Scholar Search using the HPO terms extracted from patient notes on the PhenCards patient page.**

**CITATIONS AND LICENSES**

The below information is accurate specifically at the time of this writing, licenses are subject to change.

1. This service/product is using the Human Phenotype Ontology (hpo-obo@2020-08-11). Find out more at [the HPO website](http://www.human-phenotype-ontology.org/). Citation: Sebastian Köhler, Leigh Carmody, Nicole Vasilevsky, Julius O B Jacobsen, et al. Expansion of the Human Phenotype Ontology (HPO) knowledge base and resources. Nucleic Acids Research. (2018) doi:10.1093/nar/gky1105.
2. The ICD-10 resource. Citation: World Health Organization. (2004). ICD-10 : international statistical classification of diseases and related health problems : tenth revision, 2nd ed. World Health Organization. https://apps.who.int/iris/handle/10665/42980.
3. From the ATHENA/OHDSI resource. Citation: Hripcsak G, Duke JD, Shah NH, et al. Observational Health Data Sciences and Informatics (OHDSI): Opportunities for Observational Researchers. Stud Health Technol Inform. 2015;216:574-578.
4. The Medical Subject Headings (MeSH) resource. Further information: [Pubmed Help](https://www.ncbi.nlm.nih.gov/books/NBK3827/#pubmedhelp.MeSH_Terms_MH). Citation: ROGERS FB. Medical subject headings. Bull Med Libr Assoc. 1963;51(1):114-116.
5. The Unified Medical Language System (UMLS) resource. This cannot be accessed except through the official NLM authentication API or downloaded via Metathesaurus currently. Citation: Bodenreider O. The Unified Medical Language System (UMLS): integrating biomedical terminology. Nucleic Acids Res. 2004 Jan 1;32(Database issue):D267-70. doi: 10.1093/nar/gkh061. PubMed PMID: 14681409; PubMed Central PMCID: PMC308795.
6. The Columbia Open Health Data (COHD) resource. This is freely available under the [APACHE 2.0 License](http://www.apache.org/licenses/LICENSE-2.0.html). Citation: Ta, Casey N.; Dumontier, Michel; Hripcsak, George; P. Tatonetti, Nicholas; Weng, Chunhua (2018): Columbia Open Health Data, a database of EHR prevalence and co-occurrence of conditions, drugs, and procedures. figshare. Collection. https://doi.org/10.6084/m9.figshare.c.4151252.v1
7. The Pharos database is the user interface for obtaining drug target, gene ontology, expression and pathway data for diseases, and ligand/PPI data for drug targets. This is public domain courtesy of NCATS. Sheils, T., Mathias, S. et al, "TCRD and Pharos 2021: mining the human proteome for disease biology", Nucl. Acids Res., 2021. DOI: 10.1093/nar/gkaa993
8. The Disease Ontology (DO) database. This is public domain (CC0 1.0 license). Citation: Schriml, L. M., Arze, C., Nadendla, S., Chang, Y. W., Mazaitis, M., Felix, V., Feng, G., & Kibbe, W. A. (2012). Disease Ontology: a backbone for disease semantic integration. Nucleic acids research, 40(Database issue), D940–D946. https://doi.org/10.1093/nar/gkr972.
9. Phen2Gene and the H2GKB resources. This is [MIT license](https://github.com/WGLab/Phen2Gene/blob/master/LICENSE). Citation: Zhao M., Havrilla J. M., Fang L., Chen Y., Peng J., Liu C., Wu C., Sarmady M., Botas P., Isla J., Lyon G., Weng C., Wang K. (2020). Phen2Gene: Rapid Phenotype-Driven Gene Prioritization for Rare Diseases. NAR Genomics and Bioinformatics, 2(2), lqaa032. doi:10.1093/nargab/lqaa032.
10. Pathway Commons resource. This is [CC BY 4.0 license](https://creativecommons.org/licenses/by/4.0/). Citation: Cerami, E. G., Gross, B. E., Demir, E., Rodchenkov, I., Babur, O., Anwar, N., Schultz, N., Bader, G. D., & Sander, C. (2011). Pathway Commons, a web resource for biological pathway data. Nucleic acids research, 39(Database issue), D685–D690. https://doi.org/10.1093/nar/gkq1039.
11. The 2021 DrugCentral resource. This is a [CC BY 4.0 SA license](https://drugcentral.org/privacy). Paper here: [2021 NAR paper](https://fis.fda.gov/extensions/FPD-QDE-FAERS/FPD-QDE-FAERS.html).
12. The FDA Adverse Event Reporting System (FAERS) resource for 2020. This is public domain. More about: [see here](https://academic.oup.com/nar/article/49/D1/D1160/5957163).
13. The PubMed database. This is public domain. Citation: Sayers E. The E-utilities In-Depth: Parameters, Syntax and More. 2009 May 29 [Updated 2018 Oct 24]. In: Entrez Programming Utilities Help [Internet]. Bethesda (MD): National Center for Biotechnology Information (US); 2010-. Available from: https://www.ncbi.nlm.nih.gov/books/NBK25499/.
14. The IRS 990 AWS resource. This is [CC BY 4.0 license](https://creativecommons.org/licenses/by/4.0/). This can be freely used and is available courtesy of the Internal Revenue Service.
15. The Open990 resource. This is [CC NC BY 4.0 license](https://creativecommons.org/licenses/by-nc/4.0/legalcode). This data cannot be used commercially.
16. The grant and funding information from the NIH. This comes from the Office of Extramural Research (OER) at NIH and is public domain.
17. The Federal Reporter. This displays publicly funded grants and is thus public domain courtesy of several branches of government, including the NSF and NIH.
18. Direct2Experts. The network is designed to be "open and accessible to anyone." Citation:Weber GM, Barnett W, Conlon M, Eichmann D, Kibbe W, Falk-Krzesinski H, Halaas M, Johnson L, Meeks E, Mitchell D, Schleyer T, Stallings S, Warden M, Kahlon M; Direct2Experts Collaboration. Direct2Experts: a pilot national network to demonstrate interoperability among research-networking platforms. J Am Med Inform Assoc. 2011 Dec;18 Suppl 1(Suppl 1):i157-60. doi: 10.1136/amiajnl-2011-000200. Epub 2011 Oct 28. PMID: 22037890; PMCID: PMC3241163.
19. ClinicalTrials.gov. This is public domain, courtesy of the U.S. National Library of Medicine.
20. Doc2HPO natural language processing resource. This is [CC BY 4.0 license](https://creativecommons.org/licenses/by/4.0/). Citation: Liu C., Peres Kury F. S., Li Z., Ta C., Wang K., & Weng C. (2019). Doc2Hpo: a web application for efficient and accurate HPO concept curation. Nucleic Acids Research, 47(W1), W566–W570. doi:10.1093/nar/gkz386.

# **REFERENCES**

1. Hripcsak G, Duke JD, Shah NH, Reich CG, Huser V, Schuemie MJ, et al. Observational Health Data Sciences and Informatics (OHDSI): Opportunities for Observational Researchers. Stud Health Technol Inform. 2015;216:574-8.

2. Lowe HJ, Barnett GO. Understanding and using the medical subject headings (MeSH) vocabulary to perform literature searches. JAMA. 1994;271(14):1103-8.

3. Bodenreider O. The Unified Medical Language System (UMLS): integrating biomedical terminology. Nucleic Acids Res. 2004;32(Database issue):D267-70. doi: 10.1093/nar/gkh061.

4. World Health O. International Statistical Classification of Diseases and Related Health Problems: Tabular list. World Health Organization; 2004.

5. Köhler S, Carmody L, Vasilevsky N, Jacobsen JOB, Danis D, Gourdine J-P, et al. Expansion of the Human Phenotype Ontology (HPO) knowledge base and resources. Nucleic Acids Res. 2019;47(D1):D1018-D27. doi: 10.1093/nar/gky1105.

6. Schriml LM, Arze C, Nadendla S, Chang Y-WW, Mazaitis M, Felix V, et al. Disease Ontology: a backbone for disease semantic integration. Nucleic Acids Res. 2012;40(Database issue):D940-6. doi: 10.1093/nar/gkr972.

7. McKusick VA. Mendelian Inheritance in Man and its online version, OMIM. Am J Hum Genet. 2007;80(4):588-604. doi: 10.1086/514346.

8. Weinreich SS, Mangon R, Sikkens JJ, Teeuw ME, Cornel MC. Orphanet: a European database for rare diseases. Ned Tijdschr Geneeskd. 2008;152(9):518-9.

9. Firth HV, Richards SM, Bevan AP, Clayton S, Corpas M, Rajan D, et al. DECIPHER: Database of Chromosomal Imbalance and Phenotype in Humans Using Ensembl Resources. Am J Hum Genet. 2009;84(4):524-33. doi: 10.1016/j.ajhg.2009.03.010.

10. Ta CN, Dumontier M, Hripcsak G, Tatonetti NP, Weng C. Columbia Open Health Data, clinical concept prevalence and co-occurrence from electronic health records. Sci Data. 2018;5:180273. doi: 10.1038/sdata.2018.273.

11. Zhao M, Havrilla JM, Fang L, Chen Y, Peng J, Liu C, et al. Phen2Gene: rapid phenotype-driven gene prioritization for rare diseases. NAR Genom Bioinform. 2020;2(2):lqaa032. doi: 10.1093/nargab/lqaa032.

12. Kanehisa M, Goto S. KEGG: kyoto encyclopedia of genes and genomes. Nucleic Acids Res. 2000;28(1):27-30. doi: 10.1093/nar/28.1.27.

13. Cerami EG, Gross BE, Demir E, Rodchenkov I, Babur O, Anwar N, et al. Pathway Commons, a web resource for biological pathway data. Nucleic Acids Res. 2011;39(Database issue):D685-90. doi: 10.1093/nar/gkq1039.

14. Zarin DA, Tse T, Williams RJ, Califf RM, Ide NC. The ClinicalTrials. gov results database—update and key issues. N Engl J Med. 2011;364(9):852-60.

15. Kass-Hout TA, Xu Z, Mohebbi M, Nelsen H, Baker A, Levine J, et al. OpenFDA: an innovative platform providing access to a wealth of FDA's publicly available data. J Am Med Inform Assoc. 2016;23(3):596-600. doi: 10.1093/jamia/ocv153.

16. Coordinators NR. Database resources of the National Center for Biotechnology Information. Nucleic Acids Res. 2016;44(D1):D7-19. doi: 10.1093/nar/gkv1290.

17. Liu C, Peres Kury FS, Li Z, Ta C, Wang K, Weng C. Doc2Hpo: a web application for efficient and accurate HPO concept curation. Nucleic Acids Res. 2019;47(W1):W566-W70. doi: 10.1093/nar/gkz386.

18. Pollack A. Triumph for Drug to Straighten Clenched Fingers. The New York Times2010.

19. Denkler K. Surgical complications associated with fasciectomy for dupuytren's disease: a 20-year review of the English literature. Eplasty. 2010;10:e15.

20. Nguyen D-T, Mathias S, Bologa C, Brunak S, Fernandez N, Gaulton A, et al. Pharos: Collating protein information to shed light on the druggable genome. Nucleic Acids Res. 2017;45(D1):D995-D1002. doi: 10.1093/nar/gkw1072.

21. Miller N, Lacroix EM, Backus JE. MEDLINEplus: building and maintaining the National Library of Medicine's consumer health Web service. Bull Med Libr Assoc. 2000;88(1):11-7.

22. Avram S, Bologa CG, Holmes J, Bocci G, Wilson TB, Nguyen D-T, et al. DrugCentral 2021 supports drug discovery and repositioning. Nucleic Acids Res. 2021;49(D1):D1160-D9. doi: 10.1093/nar/gkaa997.

23. Dolk H, Wang H, Loane M, Morris J, Garne E, Addor M-C, et al. Lamotrigine use in pregnancy and risk of orofacial cleft and other congenital anomalies. Neurology. 2016;86(18):1716-25. doi: 10.1212/WNL.0000000000002540.
